# Zero-Waste Conversion of *Juglans regia* and *Allium sativum* Biomass into Porous Carbons for Dye Removal and Recovery from Water

**DOI:** 10.3390/molecules31152678

**Published:** 2026-07-31

**Authors:** Nevena Radivojević, Tamara Terzić, Tamara Lazarević-Pašti, Igor Pašti, Nebojša Potkonjak, Aleksa Luković, Jasmina Mušović, Vedran Milanković

**Affiliations:** 1 Vinča Institute of Nuclear Sciences—National Institute of the Republic of Serbia, University of Belgrade, Mike Petrovica Alasa 12–14, 11000 Belgrade, Serbia; nevena.arsenijevic@vin.bg.ac.rs (N.R.); tamara.tasic@vin.bg.ac.rs (T.T.); tamara@vin.bg.ac.rs (T.L.-P.); npotkonjak@vin.bg.ac.rs (N.P.); aleksa.lukovic@vin.bg.ac.rs (A.L.); jasmina.musovic@vin.bg.ac.rs (J.M.); 2 Faculty of Physical Chemistry, University of Belgrade, Studentski Trg 12–16, 11158 Belgrade, Serbia; igor@ffh.bg.ac.rs; 3 Serbian Academy of Sciences and Arts, Kneza Mihaila 35, 11000 Belgrade, Serbia

**Keywords:** biomass-derived carbon materials, microporous structure, adsorption mechanisms, cationic dye removal, structure-property relationship, dynamic adsorption, regeneration and reuse

## Abstract

Biomass-derived porous carbons are promising sustainable adsorbents for wastewater treatment. However, most reported materials require chemical activation, while the relationships between biomass precursor, pore structure, adsorption mechanism, and regeneration remain insufficiently understood. In this work, non-activated carbon materials were prepared from *Juglans regia* (JR) and *Allium sativum* (AS) biomass by pyrolysis at 400 and 900 °C and evaluated for the removal of methylene blue (MB), rhodamine B (RB), crystal violet (CV), and malachite green (MG). Carbonization at 900 °C markedly enhanced porosity, yielding a surface area of 790 m^2^ g^−1^ for JR900 and 177 m^2^ g^−1^ for AS900, together with predominantly microporous structures and negatively charged surfaces at neutral pH. The pseudo-second-order model best described adsorption kinetics, while intraparticle diffusion analysis indicated a multistep adsorption process. Equilibrium data were well fitted by both Langmuir and Freundlich isotherm models. JR900 exhibited the highest adsorption capacities for MB (321 mg g^−1^) and RB (304 mg g^−1^), whereas AS900 showed superior performance toward MG (278 mg g^−1^). Stable dynamic filtration, efficient regeneration, and nearly complete dye recovery demonstrate the potential of these non-activated biomass-derived carbons for sustainable dye removal and recovery from water.

## 1. Introduction

The increasing release of synthetic dyes into aquatic environments represents a significant technological challenge due to their high chemical stability, toxicity, and resistance to biodegradation [1]. Cationic dyes such as methylene blue (MB), rhodamine B (RB), crystal violet (CV), and malachite green (MG) are extensively used worldwide because of their excellent coloring properties, chemical stability, and cost-effectiveness. MB is widely applied in textile dyeing and medicine [2], RB in textile, paper, and printing industries [3], and CV in textile dyeing, microbiological staining, and veterinary applications [4], while MG has been used in the textile industry and aquaculture despite increasing restrictions due to its toxicity [5]. It is estimated that approximately 10–20% of the dyes used during industrial processes are discharged into wastewater, leading to severe contamination of aquatic ecosystems [6,7]. The persistence of these pollutants in water bodies emphasizes the urgent need for effective, sustainable, and economically viable treatment strategies, aligning with SDG 6: Clean Water and Sanitation, which aims to ensure availability and sustainable management of water for all [8].

Various physicochemical and biological technologies have been developed for dye removal, including coagulation-flocculation, membrane filtration, advanced oxidation processes, photocatalysis, electrochemical treatment, and biodegradation. However, these methods are often associated with high operating costs, incomplete mineralization of pollutants, membrane fouling, excessive energy consumption, or the generation of secondary waste streams [9].

Among various treatment methods, adsorption has emerged as one of the most effective and versatile approaches due to its operational simplicity, high removal efficiency, adaptability to different pollutants, and reduced generation of secondary waste. Compared with many conventional treatment technologies, adsorption offers several important advantages, including high efficiency even at low dye concentrations, simple equipment requirements, easy operation, the possibility of adsorbent regeneration, and potential recovery of valuable adsorbates, making it particularly attractive for sustainable wastewater treatment [10]. The success of adsorption processes depends critically on the physicochemical properties of the adsorbent, such as surface area, pore structure, surface charge, and functional groups capable of interacting with dye molecules [11,12]. Carbon-based materials, especially those derived from biomass, have attracted growing attention as low-cost, renewable, and environmentally friendly adsorbents [13]. Agricultural residues, food processing by-products, and other biowaste streams can be transformed into porous carbons with tunable textural and chemical properties [14], providing a sustainable route for waste valorization within circular economy frameworks and contributing to SDG 12: Responsible Consumption and Production [8]. These bio-derived carbons often retain oxygen- and nitrogen-containing functional groups, which enhance adsorption through electrostatic interactions, hydrogen bonding, and π-π stacking [15]. Moreover, their regeneration and reusability significantly increase the feasibility of practical wastewater treatment applications, reducing waste generation and promoting resource efficiency [16].

While chemical activation is commonly used to enhance surface area and porosity, it often involves hazardous reagents, such as KOH, H_3_PO_4_, or ZnCl_2_ [17,18], and increases environmental and economic costs [19,20]. Therefore, non-activated biomass-derived carbons prepared through simple thermal treatment represent a promising and greener alternative. Achieving high surface area and well-developed porosity without chemical activation remains challenging and is strongly influenced by the nature of the precursor and carbonization temperature. Furthermore, adsorption performance is also governed by the molecular characteristics of dyes, including size, planarity, and aromaticity, which influence accessibility to micropores and the strength of interactions with the carbon surface [21]. Recent studies have demonstrated that biomass-derived porous carbons prepared from agricultural residues, fruit shells, wood wastes, and food-processing by-products exhibit excellent adsorption performance toward organic dyes [22]. However, in the vast majority of these studies, high adsorption capacities have been achieved through chemical activation using agents such as KOH, H_3_PO_4_, or ZnCl_2_, whereas non-activated carbons remain considerably less explored. Moreover, recent investigations have primarily focused on maximizing adsorption capacity toward individual model dyes, while considerably less attention has been devoted to understanding adsorption selectivity, pore–molecule size matching, dynamic filtration performance, and adsorbent regeneration within a single systematic study. By using renewable biomass as a precursor, this approach contributes to SDG 13: Climate Action by reducing emissions associated with synthetic adsorbent production and waste disposal and to SDG 14: Life Below Water by preventing toxic pollutants from entering aquatic ecosystems [8].

As already mentioned, numerous studies have reported the preparation of biomass-derived carbon adsorbents for dye removal, and the majority focus on chemically activated materials or evaluate adsorption using a single dye under batch conditions [22]. Several important knowledge gaps remain. First, the adsorption behavior of non-activated biomass-derived carbons has received significantly less attention than that of chemically activated counterparts. Second, the combined influence of biomass precursor, carbonization temperature, pore architecture, and dye molecular characteristics on adsorption selectivity has not been systematically established. Third, dynamic filtration experiments together with dye recovery and adsorbent regeneration, which are essential for practical implementation, are rarely integrated with conventional equilibrium adsorption studies. Comparative investigations that systematically correlate biomass precursor, carbonization temperature, pore structure, dye molecular characteristics, adsorption mechanisms, dynamic filtration performance, and adsorbent regeneration remain scarce. In particular, the role of pore–molecule size matching in governing adsorption selectivity in non-activated porous carbons has received relatively limited attention [23]. These limitations highlight the need for integrated studies that simultaneously evaluate material synthesis, structure-property relationships, adsorption mechanisms, and practical application under realistic operating conditions.

The aim of this study was to prepare non-activated porous carbon materials from *Juglans regia* (young walnut) and *Allium sativum* (garlic) biomass via simple thermal carbonization and to systematically investigate the relationship between precursor type, carbonization temperature, material properties, and adsorption performance toward structurally different cationic dyes under both batch and dynamic filtration conditions. *Juglans regia* and *Allium sativum* were selected as carbon precursors due to their abundance, renewable nature, and suitable lignocellulosic composition. Both crops are globally cultivated and characterized by significant annual production, ensuring their continuous availability as renewable biomass resources. These biomasses contain significant amounts of carbon-rich organic components, including cellulose, hemicellulose, and lignin, which can be converted into stable carbon frameworks through thermochemical treatment [24,25]. Therefore, these materials were considered promising precursors for the preparation of porous carbon adsorbents for water purification applications. The materials were comprehensively characterized for morphology, surface chemistry, structure, and textural properties. Their adsorption performance toward MB, RB, CV, and MG was evaluated through kinetic and equilibrium studies, including dynamic filtration experiments to simulate practical conditions. Beyond conventional surface-area-driven design, this work demonstrates that adsorption performance in non-activated carbons is governed by pore-molecule size matching, enabling selective uptake and revealing the limitations of purely capacity-based evaluation. Regeneration and reusability were systematically assessed to demonstrate not only the stability of adsorption performance, but also nearly complete recovery of all dye components, enabling a closed adsorption–desorption cycle rather than simple contaminant removal. This approach extends the role of adsorption from a separation technique to a recovery-oriented process. The novelty of this work lies in preparing high-performance porous carbons through a simple carbonization process without chemical activation and in establishing a direct relationship between biomass precursor, carbonization temperature, pore structure, and dye molecular characteristics under both equilibrium and dynamic filtration conditions. Furthermore, this study demonstrates efficient adsorbent regeneration together with dye recovery, highlighting the potential of non-activated biomass-derived carbons for sustainable and circular water treatment technologies. By transforming abundant biomass waste into high-performance carbon adsorbents, this work emphasizes the valorization of bioresources, contributes to circular economy strategies, and demonstrates a sustainable approach to wastewater treatment. The results provide new insights into the design of non-activated, biomass-derived carbon materials with strong adsorption capacity, high regeneration potential, and relevance to multiple Sustainable Development Goals (6, 12, 13, and 14).

## 2. Results and Discussion

### 2.1. Materials Characterization

**Figure 1:**
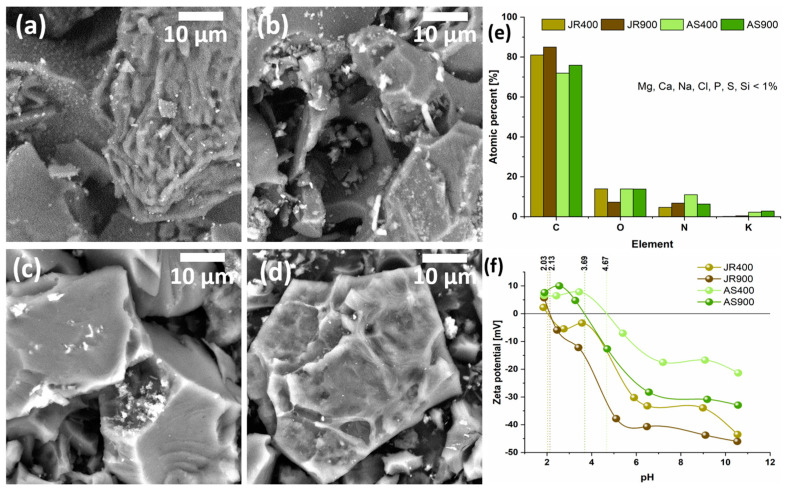
SEM micrographs (5000 × magnification) of (**a**) AS400, (**b**) AS900, (**c**) JR400, and (**d**) JR900; (**e**) Histogram representing elemental composition of all materials; (**f**) Dependence of zeta potential on pH value for all materials with indicated isoelectric points.

Figure 1 summarizes the morphological, elemental, and surface charge characterization of the synthesized JR and AS materials obtained at 400 and 900 °C. The SEM micrographs (Figure 1a–d) reveal noticeable morphological differences between the samples and a clear influence of the carbonization temperature on the surface structure. The JR400 sample exhibits a relatively compact, heterogeneous morphology, with agglomerated particles and limited visible porosity. In contrast, JR900 shows a more fragmented and porous structure, with cavities and partially collapsed layers, indicating extensive thermal decomposition and volatilization of organic components at higher temperatures [26]. A similar trend can be observed for the AS materials. The AS400 sample displays relatively smooth, plate-like structures with compact surfaces, while AS900 shows a more irregular, textured morphology with the appearance of surface defects and voids, suggesting structural rearrangement and the formation of a more developed porous structure during carbonization at 900 °C [27].

The elemental composition of all materials (Figure 1e) confirms that carbon is the dominant element, with contents ranging from approximately 70 to 85 at.%. For the JR materials, nitrogen content increases with increasing carbonization temperature, while oxygen content decreases, indicating progressive carbonization and structural rearrangement of the biomass-derived matrix. In contrast, the AS materials show no noticeable decrease in oxygen content with increasing temperature, whereas the sample treated at 900 °C shows a reduction in nitrogen content. This behavior suggests different thermal transformation pathways for the two precursors during carbonization. Other elements, such as Mg, Ca, Na, Cl, P, S, and Si, are detected only at trace levels (<1 at. %), which can be attributed to mineral residues inherited from the original biomass.

The surface charge properties of the materials were further investigated by zeta potential measurements as a function of pH (Figure 1f). All samples exhibit positive or near-neutral surface charge under strongly acidic conditions, while the zeta potential becomes increasingly negative with increasing pH. This behavior can be attributed to the protonation–deprotonation equilibria of oxygen-containing surface functional groups. Under acidic conditions, protonation of residual carboxylic, phenolic, carbonyl, and ether-type surface functionalities suppresses the negative surface charge, whereas increasing pH promotes their progressive deprotonation, resulting in increasingly negative zeta potential values. The isoelectric points are approximately pH 2.13, 2.78, 3.69, and 4.67 for JR400, JR900, AS400, and AS900, respectively. Above these pH values, the surfaces of the materials are negatively charged due to the progressive deprotonation of oxygen-containing functional groups such as carboxyl and phenolic groups. The observed trend is consistent with the FTIR results. JR400 and AS400 exhibit more pronounced bands assigned to oxygen-containing functional groups, whereas the substantially lower band intensities observed for JR900 and AS900 indicate partial removal of these functionalities during carbonization at 900 °C and the formation of a more condensed aromatic carbon structure. Nevertheless, sufficient oxygen-containing edge groups remain on the surface of the high-temperature carbons to undergo deprotonation at neutral and alkaline pH, explaining their increasingly negative surface charge. The more negative zeta potential values observed at neutral and alkaline pH indicate the predominance of deprotonated surface functionalities, which may play an important role in adsorption processes involving electrostatic interactions [28].

**Figure 2:**
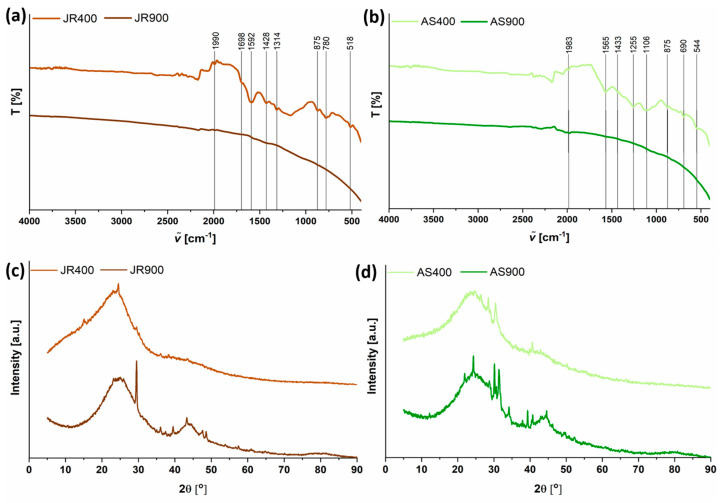
FTIR spectra (4000–400 cm^−1^) of (**a**) JR and (**b**) AS materials; XRD diffractograms of (**c**) JR and (**d**) AS materials.

The FTIR spectra recorded in the range 2000–400 cm^−1^ (Figure 2a,b) provide further insight into the surface functional groups of the materials. For JR400, several characteristic bands are observed, including signals around 1698–1592 cm^−1^ corresponding to C=O stretching and aromatic C=C vibrations [29], bands near 1428 and 1314 cm^−1^ associated with C–H deformation and C–O vibrations [30], as well as peaks at approximately 875 and 780 cm^−1^ attributed to out-of-plane aromatic C–H vibrations [31]. A band around 518 cm^−1^ may be related to inorganic mineral components. In the JR900 spectrum, the intensity of most bands significantly decreases, indicating the partial removal of oxygen-containing functional groups and the formation of a more condensed aromatic carbon structure. A similar behavior is observed for the AS materials. The AS400 spectrum shows pronounced bands at approximately 1565, 1433, 1255, and 1106 cm^−1^ corresponding to aromatic C=C vibrations and C-O stretching modes [29], along with bands at 875, 690, and 544 cm^−1^ related to aromatic structures and possible mineral phases [31]. After carbonization at 900 °C, the AS900 spectrum shows a substantial reduction in band intensity, confirming the progressive loss of functional groups and the development of a more carbonized, structurally condensed material.

The XRD patterns (Figure 2c,d) of the materials obtained at 400 °C show a broad diffraction band centered at approximately 23–25°. This broad feature indicates the presence of small and randomly oriented aromatic carbon domains with a low degree of structural ordering, which is typical for biomass-derived chars produced at relatively low carbonization temperatures [32]. After increasing the pyrolysis temperature to 900 °C, the (002) reflection becomes narrower and more defined, while a weak diffraction feature appears around 43°, which can be assigned to the (100) plane of graphitic carbon [33]. These changes indicate the development of larger aromatic domains and an increased degree of carbon ordering due to the progressive rearrangement, condensation, and aromatization of the carbon structure during high-temperature treatment. However, the persistence of broad reflections confirms that the obtained materials remain predominantly turbostratic rather than fully graphitized carbons. In addition, several sharp diffraction peaks observed in the 28–32° and 35–45° regions are attributed to crystalline inorganic phases originating from mineral constituents naturally present in the biomass. The characteristic diffraction peaks may be attributed to calcium-containing mineral phases, possibly including CaCO_3_, although definitive phase identification would require additional characterization [34].

The textural properties of the JR and AS materials obtained at 400 and 900 °C were evaluated in terms of the BET specific surface area, total pore volume, average pore diameter, and pore volume determined by NLDFT analysis. Both precursor materials exhibit very low surface areas after treatment at 400 °C. The JR400 sample shows a specific surface area of 5.59 m^2^ g^−1^ with a pore volume of 0.011 cm^3^ g^−1^, while AS400 exhibits an even lower surface area of only 1.1 m^2^ g^−1^ and a pore volume of 0.007 cm^3^ g^−1^. These results indicate that carbonization at 400 °C is insufficient to significantly develop the porous structure, consistent with the SEM observations of relatively compact, poorly developed surfaces. The average pore diameters calculated by the BET method (3.92 nm for JR400 and 12.41 nm for AS400) indicate that the limited porosity in these samples is primarily associated with larger mesopores formed by partial decomposition of the biomass structure. A substantial increase in porosity is observed for materials carbonized at 900 °C. The JR900 sample shows a dramatic increase in surface area to 790 m^2^ g^−1^ and a total pore volume of 0.467 cm^3^ g^−1^, indicating extensive pore development during high-temperature treatment. Similarly, the AS900 sample exhibits a significant increase in surface area to 177 m^2^ g^−1^ and a pore volume of 0.145 cm^3^ g^−1^. The much higher surface area of JR900 compared to AS900 suggests that the JR precursor is more favorable for forming a highly porous carbon structure under the applied conditions. The average pore diameters for JR900 (1.18 nm) and AS900 (1.64 nm) indicate that the porosity of these materials is predominantly in the microporous range. This is further supported by the NLDFT analysis, which yields higher pore volume values (0.698 cm^3^ g^−1^ for JR900 and 0.183 cm^3^ g^−1^ for AS900) compared to the BET-derived pore volumes, reflecting the presence of a significant fraction of micropores that the NLDFT model more accurately describes.

The characterization results demonstrate that the carbonization temperature plays a crucial role in determining the materials’ structural, chemical, and textural properties. Increasing the temperature from 400 to 900 °C leads to a clear transformation from compact, poorly porous structures to highly developed, fragmented, and porous morphologies, accompanied by a significant increase in specific surface area and pore volume, particularly for JR900, indicating extensive micropore formation. Elemental analysis and FTIR results confirm progressive carbonization, reflected in the reduction of oxygen-containing functional groups and the formation of a more condensed aromatic carbon structure, while differences between JR and AS suggest distinct thermal transformation pathways of the precursors. The XRD analysis further supports the transition from predominantly amorphous carbon to a more ordered, partially graphitic structure at higher temperatures, along with the presence of residual mineral phases. Zeta potential measurements reveal that all materials become negatively charged at neutral and alkaline pH, highlighting the role of deprotonated surface functional groups in potential adsorption mechanisms.

### 2.2. Effect of Adsorption Conditions

**Figure 3:**
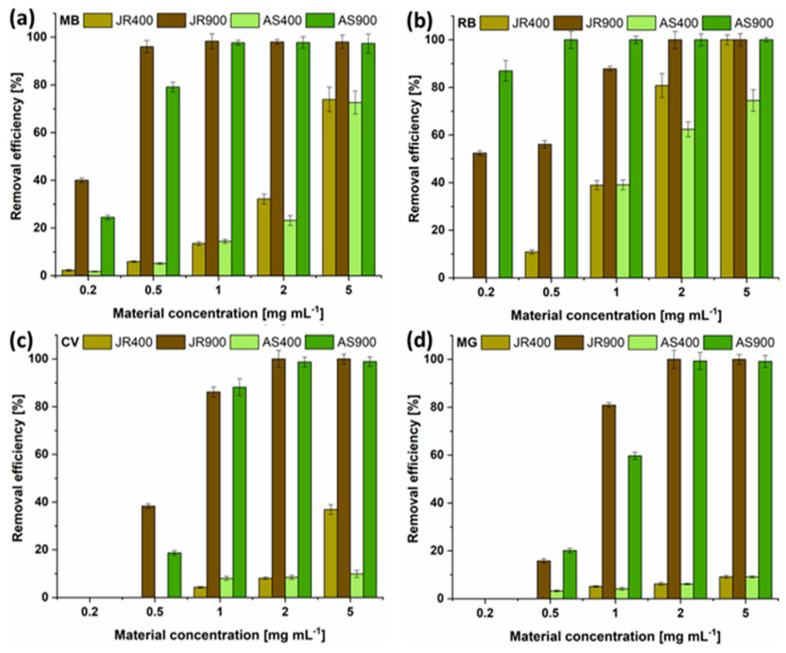
Dependence of removal efficiency of (**a**) MB, (**b**) RB, (**c**) CV, and (**d**) MG on the investigated materials of material concentration.

**Figure 4:**
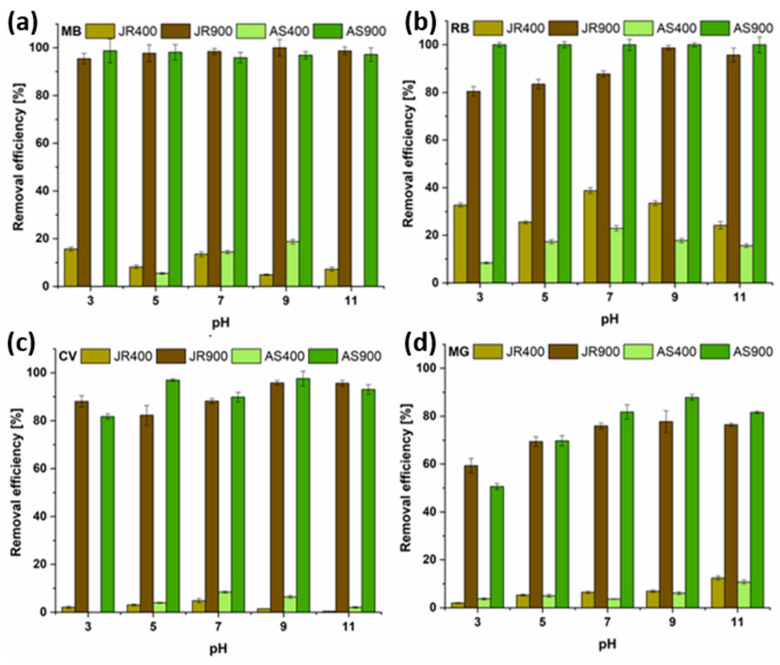
Dependence of removal efficiency of (**a**) MB, (**b**) RB, (**c**) CV, and (**d**) MG on the investigated materials of pH.

**Figure 5:**
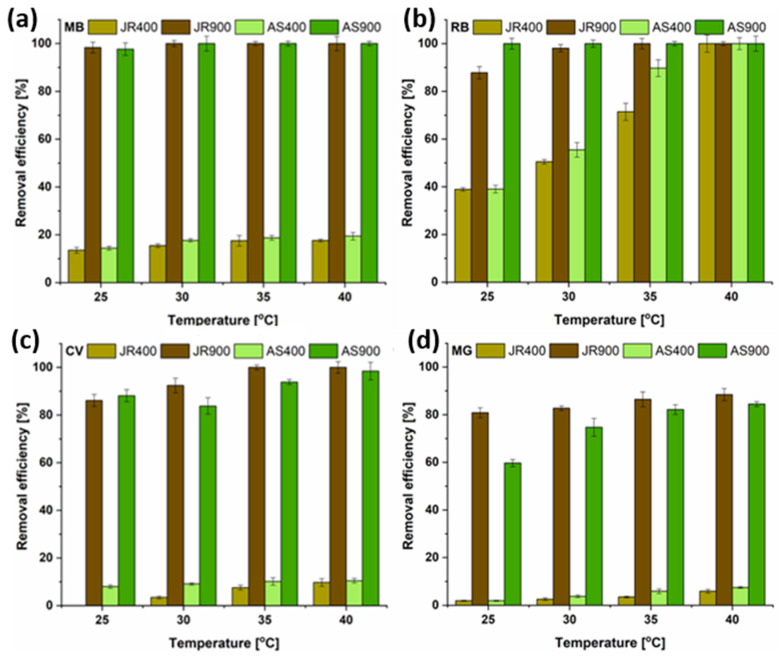
Dependence of removal efficiency of (**a**) MB, (**b**) RB, (**c**) CV, and (**d**) MG on the investigated materials and adsorption temperature.

Figure 3, Figure 4 and Figure 5 show the influence of material concentration, pH, and temperature on the removal efficiency of four model cationic dyes (MB, RB, CV, and MG) using the investigated materials. The obtained results were used as a preliminary screening to identify the most suitable experimental conditions for further adsorption studies.

A strong dependence of removal efficiency on the material concentration can be observed for all dyes (Figure 3). In general, increasing the adsorbent concentration leads to a significant increase in removal efficiency due to the greater availability of active adsorption sites. However, the extent of this increase strongly depends on the material. The materials obtained at 400 °C exhibit low removal efficiencies (0–36.9%) for MB (2.30–32.2%), RB (0–80.8%), CV (0–8.08%), and MG (0%) CV and MG across the concentration range 0.2–2 mg mL^−1^, consistent with their poorly developed porous structure and very low specific surface areas (5.59 m^2^ g^−1^ JR400 and 1.1 m^2^ g^−1^ AS400) observed in the BET analysis. Statistical analysis confirmed significant differences among the investigated adsorbent concentrations for most adsorbent–dye systems (Table S1, Supplementary Materials), demonstrating that increasing the adsorbent dosage significantly enhanced dye removal. In contrast, JR900 and AS900 show markedly higher removal efficiencies (≈100%) even at 2 mg mL^−1^. According to the statistical analysis, increasing the adsorbent concentration beyond 1 mg mL^−1^ generally resulted in no further significant improvement for JR900 and AS900, whereas more pronounced concentration-dependent effects were observed for JR400 and AS400. Based on these observations, a concentration of 1 mg mL^−1^ was selected as a suitable compromise between adsorption efficiency and material consumption.

The effect of pH on dye removal is presented in Figure 4. The adsorption efficiency generally increases with increasing pH, particularly for the cationic dyes CV and MG. This behavior is consistent with the zeta potential results (Figure 1f), which show that the materials’ surfaces become increasingly negatively charged above their isoelectric points. Under such conditions, electrostatic attraction between the negatively charged adsorbent surface and the positively charged dye molecules becomes stronger, thereby improving adsorption. Statistical analysis confirmed that pH significantly influenced the adsorption performance of most adsorbent-dye systems (Table S2, Supplementary Materials), although the magnitude of this effect depended on both the adsorbent and the dye. Although the highest removal efficiencies are often observed at alkaline pH values, a neutral pH of 7 was selected for further experiments. The statistical analysis further indicated that pH 7 provided adsorption efficiencies comparable to those obtained under more alkaline conditions for the most efficient adsorbents, while better representing environmentally relevant conditions. This pH lies within the common range of natural waters (≈6–8) and allows evaluation under conditions that minimize chemical adjustment, thereby improving its relevance to practical water treatment scenarios.

The influence of temperature on removal efficiency is shown in Figure 5. In general, a moderate increase in removal efficiency with increasing temperature is observed in some systems, which may indicate contributions from enhanced diffusion or slightly endothermic adsorption processes. Statistical analysis confirmed that temperature affected the adsorption performance of most adsorbent–dye systems (Table S3, Supplementary Materials), although the extent of this effect varied depending on the adsorbent and dye. However, the differences between adsorption at 25 and 40 °C are relatively small for most dye-adsorbent combinations, especially for the high-performing materials JR900 and AS900, which already show very high removal efficiencies at 25 °C (>80%, except for MG adsorption onto AS900 (≈60%)). Considering both the statistical analysis and the limited practical improvement achieved at elevated temperatures, 25 °C was selected as the reference temperature for all subsequent experiments, allowing consistent comparison of adsorption performance without additional energy input.

Consequently, JR400 and AS400 were excluded from further adsorption studies due to their consistently low performance, with removal efficiencies below 50% in most cases. Based on the combined analysis of concentration, pH, and temperature effects, pH 7, an adsorbent concentration of 1 mg mL-1, and 25 °C were selected as reference conditions for consistent comparison of adsorption performance under controlled experimental conditions.

### 2.3. Kinetic and Isotherm Study of the Adsorption of Cationic Dyes onto Investigated Materials

**Figure 6:**
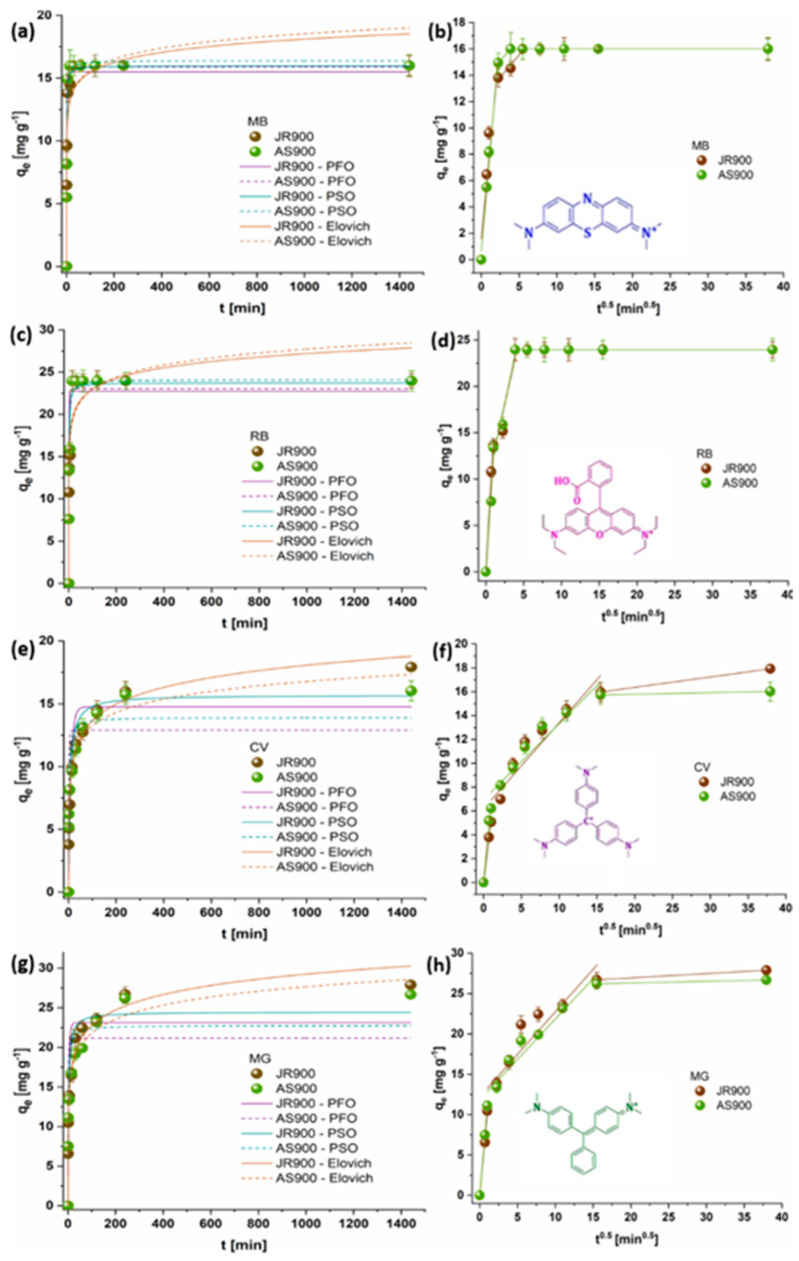
Experimental adsorption data fitting to (**a**) kinetic adsorption models for MB, (**b**) intraparticle diffusion model for MB, (**c**) kinetic adsorption models for RB, (**d**) intraparticle diffusion model for RB, (**e**) kinetic adsorption models for CV, (**f**) intraparticle diffusion model for CV, (**g**) kinetic adsorption models for MG, and (**h**) intraparticle diffusion model for MG.

**Figure 7:**
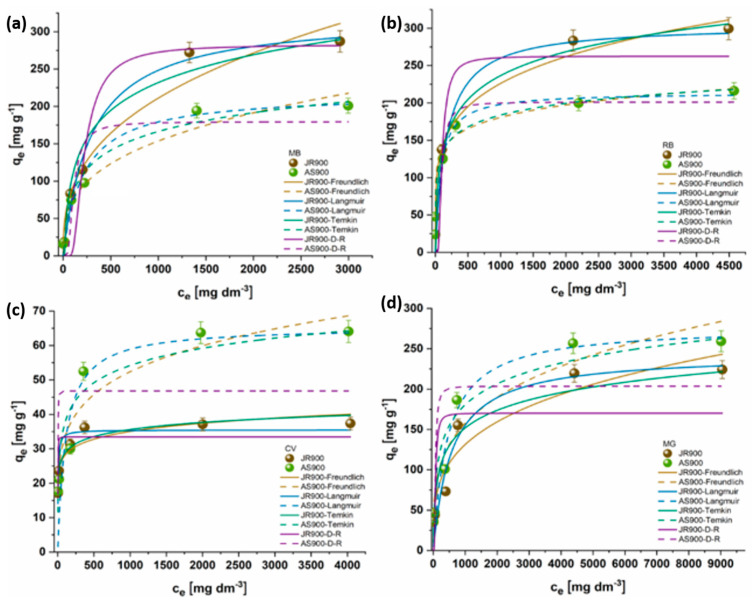
Experimental adsorption data fitting to adsorption isotherm models for (**a**) MB, (**b**) RB, (**c**) CV, and (**d**) MG.

The adsorption kinetics of four dyes onto selected materials were analyzed using the PFO, PSO, and Elovich kinetic models, and the corresponding fits to the experimental data are presented in Figure 6a,c,e,g. The IPD model is shown in Figure 6b,d,f,h. The obtained kinetic parameters for all models are summarized in Table 1. The fits of the experimental data to the Freundlich, Langmuir, Temkin, and Dubinin-Radushkevich isotherm models are presented in Figure 7, while the corresponding parameters are summarized in Table 2.

From the experimental data presented in Figure 6 (left panels), it can be concluded that the adsorption equilibria of MB and RB are reached within the first 30 min, whereas for CV and MG, it takes up to 24 h. This trend was further supported by the statistical analysis (Table S4, Supplementary Materials), which showed significant changes in adsorption capacity during the initial adsorption stage for all dye-adsorbent systems. For MB and RB, no statistically significant differences were observed after 30 min, confirming that adsorption equilibrium had been attained. In contrast, CV and MG continued to exhibit significant differences at longer contact times, indicating that equilibrium was reached only after prolonged adsorption. To ensure consistency and enable direct comparison across all systems, a contact time of 24 h was selected for all subsequent adsorption experiments. The kinetic analysis of MB, RB, CV, and MG adsorption onto JR900 and AS900 (Table 1) indicates that the PSO model provides a very good description of the experimental data. High correlation coefficients are obtained for this model, while the calculated equilibrium adsorption capacities are in good agreement with experimental values in both cases.

The rate constants reveal clear trends related to molecular size and accessibility of adsorption sites. Higher rate constants are generally observed for MB and RB, while significantly lower values are obtained for CV and MG, indicating slower uptake. This behavior is consistent with the predominantly microporous structure of both materials (d_av_ ≈ 1.18 nm for JR900 and 1.64 nm for AS900), where diffusion limitations become more pronounced for larger dye molecules.

The Elovich model provides additional insight into the adsorption process, particularly regarding surface heterogeneity and activation barriers. The parameter α, which represents the initial adsorption rate, shows very high values for MB and RB, indicating very rapid initial uptake, likely due to the availability of easily accessible surface sites. In contrast, significantly lower α values are observed for CV and MG, reflecting a much slower initial adsorption stage, which can be attributed to steric hindrance and restricted diffusion into narrow pores. The β parameter shows relatively consistent values across systems but with noticeable trends. Higher β values indicate a steeper decrease in the adsorption rate with increasing surface coverage, suggesting that adsorption sites become progressively less accessible as adsorption proceeds. Lower β values for CV and MG indicate a more gradual decrease in adsorption rate, consistent with a broader distribution of energetically diverse sites and a more pronounced influence of surface heterogeneity. The excellent fit of the Elovich model for CV and MG further indicates that heterogeneous surface properties and diffusion constraints strongly influence the adsorption of these dyes.

The intraparticle diffusion model reveals a multi-step adsorption mechanism (Figure 6, right panels). The presence of three distinct linear regions (I, II, and III) indicates that adsorption proceeds through: (I) rapid external surface adsorption or boundary layer diffusion, (II) gradual intraparticle diffusion into pores, and (III) a final equilibrium stage [35].

The first stage is characterized by high k_id_ values, confirming a fast initial uptake. However, the fact that the lines do not pass through the origin (non-zero C values) indicates that intraparticle diffusion is not the sole rate-limiting step and that film diffusion or surface adsorption also contributes significantly. The second stage shows much lower kid values, reflecting slower diffusion into the internal pore structure. The third stage, with k_id_ ≈ 0, corresponds to the equilibrium state.

Interestingly, k_id_ values in the first stage for RB (13.95 mg g^−1^ min^−0.5^ for JR900 and 12.89 mg g^−1^ min^−0.5^ for AS900) and MG (10.25 mg g^−1^ min^−0.5^ for JR900 and 11.02 mg g^−1^ min^−0.5^ for AS900) were higher compared to MB (5.97 mg g^−1^ min^−0.5^ for JR900 and 6.61 mg g^−1^ min^−0.5^ for AS900) and CV (5.13 mg g^−1^ min^−0.5^ for JR900 and 6.42 mg g^−1^ min^−0.5^ for AS900), suggesting faster diffusion into larger pores or weaker steric limitations at the initial stage. The differences between JR900 and AS900 are relatively subtle, though AS900 generally shows slightly higher diffusion constants in the first stage, suggesting a more accessible pore network.

The adsorption behavior of MB, RB, CV, and MG onto JR900 and AS900 at 25 °C can be fully interpreted only by integrating the isotherm results (Table 2) with the previously obtained physicochemical characterization. Such a combined analysis clearly indicates that a complex interplay of surface chemistry, pore structure, and structural heterogeneity of the carbon materials governs adsorption.

The isotherm analysis shows that both the Langmuir and the Freundlich models adequately describe the equilibrium data. For most systems, both models yield high correlation coefficients, indicating that adsorption cannot be attributed to a single idealized adsorption regime. Instead, the results suggest the presence of surface heterogeneity and a distribution of adsorption energies. This behavior is in excellent agreement with the XRD patterns, which reveal a partially ordered carbon structure at 900 °C, characterized by the coexistence of graphitic domains ((002) and (100) reflections) and amorphous regions. Such structural heterogeneity likely reflects a range of adsorption sites with different affinities, rather than distinct mechanistic regimes.

The maximum adsorption capacities further reflect the influence of textural properties. JR900 exhibits significantly higher capacities for MB (321 ± 4 mg g^−1^) and RB (304 ± 7 mg g^−1^) compared to AS900 (217 ± 4 and 210 ± 10 mg g^−1^, respectively), consistent with its much higher specific surface area and larger pore volume. The well-developed porous structure of JR900, characterized by its predominantly microporous character (d_av_ ≈ 1.18 nm), provides a large number of accessible adsorption sites and is consistent with the efficient uptake of smaller dye molecules such as MB. In contrast, AS900, despite its lower surface area, shows a relatively high capacity for MG (278 ± 8 mg g^−1^), suggesting that factors beyond surface area, such as pore size distribution and surface chemistry, play an important role in adsorption behavior. These differences can be attributed to distinct thermal transformation pathways of the precursor materials, leading to variations in pore architecture and surface functionality.

The Freundlich constants further support this interpretation. All n values are greater than 1 (ranging from 2.76 to 10.5), confirming favorable adsorption, while particularly high n values for CV indicate strong adsorption on specific high-energy sites [17]. Considering that adsorption experiments were performed exclusively on materials carbonized at 900 °C, where FTIR spectra show a significant loss of oxygen-containing functional groups, these high-energy sites are more likely associated with structural defects, edge sites, and π-electron-rich aromatic domains rather than polar surface functionalities. This interpretation is consistent with the XRD results, which indicate an increased degree of structural ordering alongside the presence of disordered regions, as well as with the highly developed microporosity revealed by BET analysis. Furthermore, the increasingly negative surface charge at neutral pH, as evidenced by zeta potential measurements, still enables electrostatic attraction between the carbon surface and cationic dyes despite the reduced presence of functional groups. Therefore, adsorption is predominantly governed by π-π interactions between the graphitic domains and the aromatic dye molecules, complemented by electrostatic interactions and confinement effects within the microporous structure.

The Langmuir constant K_L_ provides additional insight into adsorption affinity [36]. For example, the very high K_L_ value for CV on JR900 indicates stronger interaction, but the corresponding q_max_ is relatively low (35 ± 3 mg g^−1^). This suggests that although the interaction between CV and the surface is strong, the number of accessible adsorption sites is limited, most likely due to steric hindrance and restricted diffusion within narrow micropores, as supported by the BET and NLDFT results indicating dominant microporosity.

The Temkin model, which also shows relatively good agreement with experimental data, suggests that the heat of adsorption decreases with increasing surface coverage, implying significant adsorbate-adsorbate interactions. This behavior can be attributed to π-π stacking between aromatic dye molecules and the graphitic domains of the adsorbents, as well as possible lateral interactions between adsorbed species at higher surface coverage. In contrast, the Dubinin-Radushkevich model exhibited a poorer fit, which can be attributed to its simplified assumptions, primarily the predominance of micropore filling as the governing adsorption mechanism. Although the investigated materials possess pronounced microporosity, adsorption of the studied molecules likely involves additional contributions from mesopore filling, surface heterogeneity, and specific adsorbate-adsorbent interactions, which are not adequately described by the Dubinin-Radushkevich model.

### 2.4. Dynamic Adsorption Conditions, Regeneration, and Reuse

**Figure 8:**
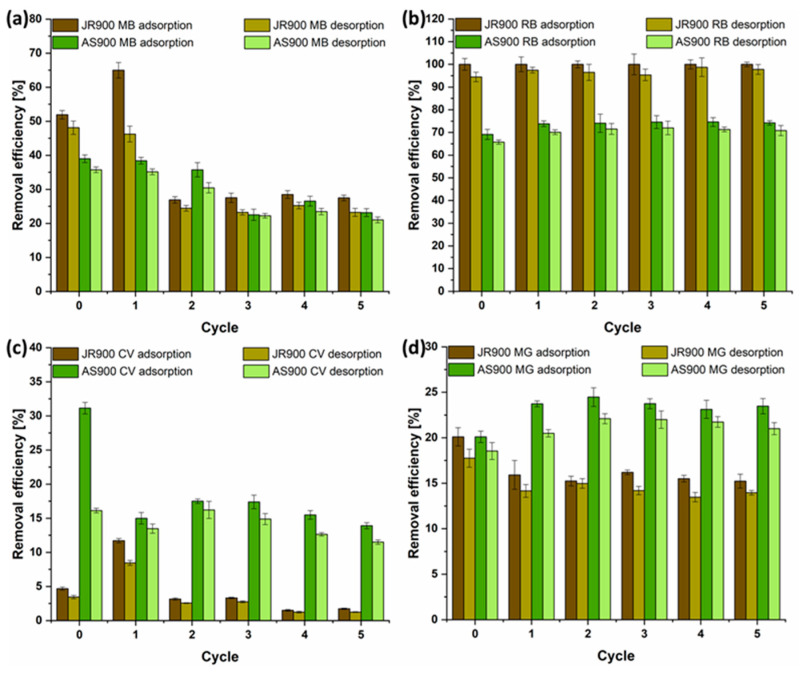
Histograms representing adsorption and desorption efficiency of (**a**) MB, (**b**) RB, (**c**) CV, and (**d**) MG during 5 cycles of regeneration.

Figure 8 illustrates the regeneration and reusability performance of JR900 and AS900 and dyes during five consecutive adsorption–desorption cycles under dynamic filtration conditions. The statistical analysis further demonstrated that the adsorption performance remained stable over repeated regeneration cycles for the most efficient dye–adsorbent systems, while systems exhibiting lower adsorption efficiencies showed only minor variations throughout the investigated cycles (Table S5, Supplementary Materials).

For MB (Figure 8a), both materials exhibit a gradual decrease in removal efficiency with an increasing number of cycles. The JR900 material initially exhibits a higher removal efficiency (50–65%), which decreases to approximately 25–30% after several cycles. AS900 follows a similar trend, but with slightly lower overall efficiencies. This decline indicates that a fraction of adsorption sites may become progressively less accessible during repeated cycles, likely due to partial pore blocking or stronger interactions between MB molecules and the carbon surface that are not completely reversed during regeneration.

In contrast, RB adsorption remains highly stable throughout the regeneration experiment (Figure 8b). JR900 maintains nearly complete removal (~100%) across all five cycles, while AS900 consistently achieves removal efficiencies around 70–75%. The nearly unchanged adsorption performance indicates that the adsorption–desorption process for RB is highly reversible and that the structural integrity of the adsorbent-packed filters remains preserved during repeated dynamic operation.

For CV (Figure 8c), the removal efficiencies are relatively low under the applied dynamic conditions. JR900 exhibits very limited adsorption (<10%) across all cycles, whereas AS900 shows higher, though still moderate, efficiencies (approximately 15–30%). Similarly, for MG (Figure 8d), both materials demonstrate relatively low but stable removal efficiencies over the regeneration cycles, with JR900 maintaining values around 15–20% and AS900 around 20–25%. These results suggest that adsorption of CV and MG under dynamic filtration conditions is likely limited by slower adsorption kinetics and insufficient contact time between the dye molecules and the adsorbent surface.

Importantly, the dyes are almost completely recovered during the washing step, as reflected in the desorption efficiency histograms (Figure 8). The characteristic absorption bands of the dyes are clearly observed in the eluents, indicating that nearly all adsorbed dye molecules are successfully desorbed from the adsorbent surface. This demonstrates not only the high efficiency of the regeneration process but also the high reversibility of the adsorption–desorption cycle. Moreover, the preservation of the characteristic absorption bands confirms that the molecular structure of the dyes remains unchanged, indicating that the dyes are recovered in their original form and can potentially be reused. The FTIR spectra of the materials after adsorption–desorption cycles (Figure S1, Supplementary Materials) show no differences after 5 cycles. Statistical analysis revealed no significant differences in adsorption efficiency over five consecutive regeneration cycles, confirming the excellent reusability of the adsorbents.

### 2.5. Discussion and Comparative Study with Other Biomass-Based Adsorbents

The adsorption behavior strongly depends not only on the molecular size of the dyes but also on their molecular geometry and steric accessibility. To better illustrate the relationship between molecular dimensions and pore structure, Table 3 compares the calculated geometric dimensions and approximate van der Waals dimensions of MB, RB, CV, and MG with the characteristic pore widths obtained by NLDFT analysis. It should be noted that the calculated molecular dimensions represent isolated molecular conformers rather than rigid kinetic diameters in aqueous solution, whereas the NLDFT values correspond to characteristic pore widths within a pore size distribution rather than uniform cylindrical pores. Therefore, the comparison should be regarded as a qualitative assessment of pore accessibility rather than a strict pore–molecule matching criterion.

MB possesses the smallest effective molecular cross-section together with a nearly planar phenothiazine structure, facilitating diffusion into the predominantly microporous structure of JR900 and resulting in high adsorption capacity. This behavior is consistent with previous studies reporting that the adsorption of MB on biomass-derived carbons is promoted by the availability of micropores and favorable π-π interactions between aromatic carbon domains and dye molecules [37]. RB exhibits the largest overall molecular envelope; however, the presence of a rotatable carboxyphenyl substituent provides considerable conformational flexibility, allowing different molecular orientations and facilitating adsorption on wider pores, pore entrances, and external adsorption sites. Consequently, its relatively large molecular dimensions do not prevent efficient adsorption.

Although CV and MG possess comparable molecular dimensions, their adsorption behavior differs markedly. CV exhibits a highly branched and relatively symmetric triphenylmethane structure containing three dimethylaminophenyl groups, producing a larger effective molecular cross-section and stronger steric constraints during diffusion through narrow pore entrances. This interpretation is fully consistent with the equilibrium isotherms, where CV exhibits an exceptionally high Langmuir affinity constant but the lowest maximum adsorption capacity, indicating strong interaction with a limited number of accessible adsorption sites. In contrast, MG contains only two dimethylaminophenyl substituents and is therefore less sterically hindered, allowing access to a larger fraction of the pore network and consequently exhibiting substantially higher adsorption capacities. These observations are consistent with previous studies demonstrating that molecular geometry and steric accessibility, rather than molecular size alone, govern the adsorption behavior of structurally different cationic dyes [38].

Under dynamic filtration conditions, the lower removal efficiencies observed for CV and MG can be attributed to the combined influence of steric hindrance, mass-transfer limitations, and restricted pore accessibility. Because the residence time within the adsorbent bed is limited, adsorption is controlled not only by equilibrium affinity but also by external mass transfer and intraparticle diffusion. Consequently, only a fraction of the available adsorption sites can be reached before the solution exits the adsorbent bed, explaining why dyes exhibiting high equilibrium adsorption capacities may still show relatively modest removal under dynamic conditions. These findings suggest that dynamic adsorption performance could be further improved by increasing the residence time through lower flow rates or greater bed depth, increasing the adsorbent loading, or designing hierarchical porous carbons containing interconnected micro- and mesopores to facilitate mass transport while maintaining high adsorption capacity. Overall, the comparison presented in Table 3 demonstrates that adsorption selectivity is governed by the combined effects of molecular geometry, steric accessibility, conformational flexibility, pore-size distribution, adsorption affinity, and mass-transfer limitations rather than molecular size alone.

**Figure 9:**
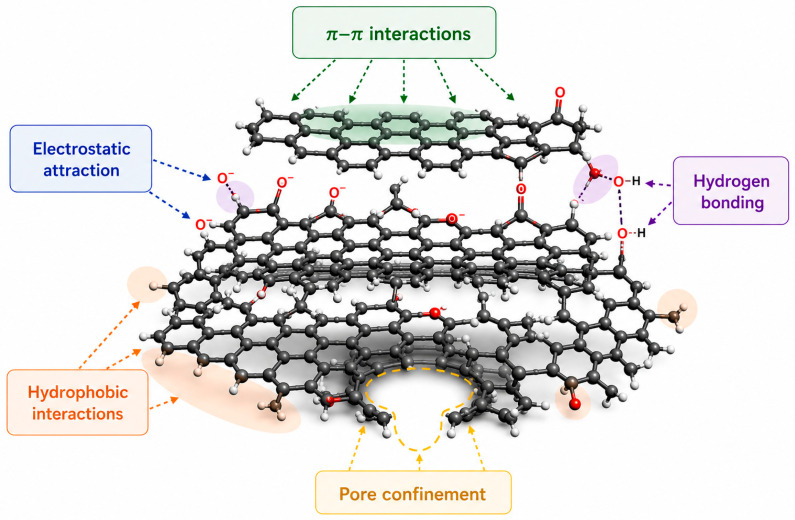
Schematic representation of the proposed adsorption mechanisms on biomass-derived carbon surfaces, including π-π interactions, electrostatic interactions, hydrogen bonding, hydrophobic interactions, and pore confinement effects.

A schematic representation of the proposed adsorption mechanisms, including π-π interactions, electrostatic interactions, hydrogen bonding, hydrophobic interactions, and pore confinement effects occurring at the carbon surface, is presented in Figure 9.

The regeneration and reusability results demonstrate that the adsorption process is largely reversible, particularly for RB, where stable performance over multiple cycles indicates minimal structural degradation and efficient desorption. Although a gradual decline in MB removal efficiency is observed, likely due to partial pore blocking or stronger binding interactions, the overall regeneration efficiency remains high. Importantly, the desorption data confirm that nearly all adsorbed dye molecules are recovered in their original form, highlighting not only the effectiveness of the regeneration procedure but also the potential for dye recovery and reuse.

To assess the practical applicability of the developed adsorbents, additional experiments were performed using real wastewater samples spiked with the investigated dyes. Comparable removal efficiencies were obtained, indicating that the wastewater matrix had no significant influence on the adsorption performance under the investigated experimental conditions. Since these results followed the same trends as those obtained in model solutions, they are not presented separately.

To further evaluate the performance of the developed materials, a comparative analysis with other biomass-derived carbon adsorbents reported in the literature was conducted (Table S6, Supplementary Materials). For MB, materials derived from spent coffee grounds carbonized at 900 °C show relatively low capacities (1.1–9.7 mg g^−1^), even when chemical (KOH, H_3_PO_4_) or physical (CO_2_) activation is applied [39]. Higher capacities are reported for carbon materials derived from *Bidens pilosa* (200 mg g^−1^ at 550 °C, without activation) [40] and seaweed (243.84 mg g^−1^ at 500 °C with NaOH activation) [41]. The materials developed in this work, derived from *Juglans regia* and *Allium sativum* and carbonized at 900 °C without additional activation, exhibit high adsorption capacities (321 and 217 mg g^−1^, respectively), placing them within the upper range of reported values.

A similar trend is observed for RB, where adsorption performance varies depending on both precursor and activation conditions. Carbon materials derived from *Atropa belladonna* carbonized at 500 °C and chemically activated with ZnCl_2_ or H_3_PO_4_ achieve capacities of 263.19 and 309.11 mg g^−1^, respectively [42], while carbons derived from *Samanea saman* (450 °C, ZnCl_2_ activation) show lower performance (101.01 mg g^−1^) [43]. In contrast, carbon materials derived from spent coffee grounds at 900 °C, adsorption capacity strongly depends on the activation: physical activation with CO_2_ results in low performance (4.2 mg g^−1^), chemical activation with KOH yields a moderate capacity (42.9 mg g^−1^), while combined chemical (KOH) and physical (CO_2_) activation significantly improves the capacity (130 mg g^−1^) [39]. The materials derived from *Juglans regia* and *Allium sativum* in this study (900 °C, no activation) achieve 304 and 210 mg g^−1^, indicating competitive performance relative to many precursor–activation combinations reported in the literature.

For CV, the highest capacities are typically associated with carbons derived from *Moringa oleifera* seed husk carbonized at 900 °C and chemically activated with H_3_PO_4_ (469.55 mg g^−1^) [44]. Other materials, such as carbons derived from cocoa leaves (700 °C, no activation) [45] and *Leonurus cardiaca* (low-temperature carbonization 105 °C) [46], exhibit moderate capacities (≈176–253 mg g^−1^). In comparison, the materials derived from *Juglans regia* and *Allium sativum* in this work (900 °C, no activation) show lower capacities (35 and 65 mg g^−1^), suggesting a lower affinity toward CV under the applied conditions.

For MG, adsorption capacities reported in the literature vary widely, depending on both the precursor and the activation method. Carbons derived from *Livistona chinensis* carbonized at 500 °C without activation exhibit low capacity (21.41 mg g^−1^) [47], while carbons derived from *Rumex abyssinicus* under similar conditions (500 °C, no activation) show improved performance (98.43 mg g^−1^) [48], highlighting the influence of precursor chemistry. Further enhancement is observed for carbon materials derived from *Dodonaea viscosa* and *Ziziphus jujube* [49], both carbonized at 800 °C and chemically activated with NaHCO_3_, achieving capacities of 208.05 and 226.3 mg g^−1^, respectively. In comparison, the materials derived from *Juglans regia* and *Allium sativum* in this study (900 °C, no activation) exhibit capacities of 243 and 278 mg g^−1^, respectively, which are comparable to or higher than those of many chemically activated systems.

Overall, when adsorption performance is considered alongside precursor origin and synthesis conditions, the materials derived from *Juglans regia* and *Allium sativum* demonstrate consistently strong performance for MB, RB, and MG, despite the absence of chemical activation, while showing more limited efficiency for CV. These observations highlight the combined influence of precursor chemistry and resulting surface properties on dye-specific adsorption behavior.

### 2.6. Economic and Practical Considerations

The economic attractiveness of the proposed adsorbents arises primarily from the use of abundant biomass as carbon precursors and the omission of chemical activation. Unlike conventional activated carbons, which commonly require activating agents such as KOH, H_3_PO_4_, or ZnCl_2_ followed by extensive washing and neutralization steps, the materials developed in this study were produced solely by thermal carbonization. Consequently, chemical consumption, wastewater generation, and post-treatment requirements are substantially reduced [50]. The main energetic cost of the synthesis is associated with the carbonization step at 900 °C. Although high-temperature treatment requires energy input, it represents a single processing step without subsequent activation or chemical regeneration of the material. Furthermore, the carbonization process can be readily integrated with continuous pyrolysis technologies and supplied by renewable electricity or energy recovered from biomass pyrolysis gases, reducing the overall environmental and economic burden [51]. Compared with commercial activated carbons, the present materials exhibit lower production complexity because they eliminate the activation stage while maintaining competitive adsorption performance toward cationic dyes, particularly MB and RB. In addition, the demonstrated regeneration and dye recovery over repeated adsorption–desorption cycles further improve the practical feasibility of the proposed adsorbents by extending their service life and reducing material consumption [52].

## 3. Materials and Methods

### 3.1. Materials Synthesis and Characterization

In this work, two different precursors were employed for the synthesis of carbon materials. One of the precursors was young walnut (*Juglans regia*), while the other was garlic (*Allium sativum*). Both were bought in the local market. The precursors were dried in a drying oven (Instrumentaria ST-05, Instrumentaria, Zagreb, Croatia) at 90 °C for 24 h. The carbonization process was carried out in an electric tubular furnace (Protherm Furnaces, Ankara, Turkey) under an inert nitrogen atmosphere. The temperature was increased from room temperature at a heating rate of 5 °C min^−1^ up to 400 or 900 °C, after which the process was maintained at a constant temperature for 1 h. The carbonization temperatures of 400 and 900 °C were selected to represent mild and severe thermal treatment conditions, respectively, enabling the evaluation of how increasing carbonization severity affects the development of carbon structure, surface chemistry, porosity, and adsorption performance. After carbonization, the furnace was allowed to cool to room temperature under a nitrogen atmosphere, and the obtained carbon materials were removed from the furnace. The carbonized products were manually ground for 30 min and subsequently sieved through a 100 µm mesh (Cisa Cedaceria Industrial S.L., Barcelona, Spain) to obtain a homogeneous powder. For purification, 100 mg of the ground carbon material was sequentially washed with 50 mL of 0.1 mol dm^−3^ NaOH, 0.1 mol dm^−3^ HCl, and deionized water (Milli-Q water purification system, Merck KGaA, Darmstadt, Germany; resistivity 18.2 MΩ) to remove residual inorganic impurities. After each washing step, the suspension was centrifuged at 6000 rpm for 15 min, and the supernatant was carefully separated from the solid phase. Washing with deionized water was repeated until the pH of the supernatant reached approximately 7. The purified carbon materials were finally dispersed in deionized water for further use.

The chemicals used in the study were of analytical grade: NaOH (≥98%, Centrohem, Stara Pazova, Serbia), HCl (37%, Centrohem, Stara Pazova, Serbia), and ethanol (≥99.8%, Merck, Darmstadt, Germany).

Morphology and surface structure of the material were investigated using scanning electron microscopy (SEM) with a Phenom ProX microscope, in combination with energy-dispersive X-ray spectroscopy (EDX), which was employed to determine the elemental composition and spatial distribution of elements (Thermo Fisher Scientific, Waltham, MA, USA). FTIR spectroscopy (Nicolet iS20, Thermo Fisher Scientific, Waltham, MA, USA) equipped with an ATR was used to analyze surface functional groups and molecular bonding. Spectra were recorded in the range of 4000–400 cm^−1^ by averaging 64 scans at a resolution of 4 cm^−1^.

The zeta potential of the adsorbents was measured using a Nano ZS Zetasizer (Malvern Instruments, Malvern, UK) equipped with a He-Ne laser (633 nm). Each reported value represents the average of three independent measurements.

XRD analysis was employed to determine the structural properties of the synthesized materials. Measurements were carried out using a Rigaku Ultima IV diffractometer equipped with CuKα1,2 radiation (Tokyo, Japan). The instrument was operated at 40 kV and 40 mA. Diffraction patterns were recorded over a 2θ range of 5–90° with a step size of 0.02° and a scan speed of 5° min^−1^, using a D/Tex digital detector for data acquisition. Phase identification was performed using PDXL2 software (version 2.8.3.0) in combination with the International Center for Diffraction Data (ICDD) database [53].

Textural parameters, including specific surface area (S_BET_), total pore volume (V_tot_), and pore size distribution (PSD), were determined from nitrogen adsorption isotherms at −196.15 °C using a BELSORP-MINI X gas sorption analyzer (MicrotracBEL, Osaka, Japan). The BET method was applied to estimate surface areas, while the NLDFT model was used to assess PSD. Before the analysis, the samples were degassed under vacuum at 300 °C for 8 h to eliminate residual adsorbates on the surface and warrant reliable measurements.

### 3.2. Adsorption Experiments

MB, RB, CV, and MG (Sigma-Aldrich, Søborg, Denmark, purity ≥ 90%) were used as model cationic dyes without further purification. All solutions were prepared using deionized water. Stock dye solutions were prepared by dissolving appropriate amounts of each dye in deionized water. Working solutions of desired concentrations were obtained by successive dilution. Each operational parameter was individually varied to isolate its effect on dye adsorption, while all other conditions were kept constant (pH 7, temperature 25 °C, adsorbent concentration 1 mg mL^−1^, and contact time 60 min, unless otherwise stated). The effect of pH was examined over the range of 3–11, temperature was varied from 25 to 40 °C, adsorbent concentration from 0.2 to 5 mg mL^−1^, and contact time from 1 min to 24 h. A summary of the experimental conditions for the adsorption experiments is given in Table 4.

Batch adsorption experiments were performed by mixing appropriate volumes of stock suspensions of the adsorbents with stock dye solutions (final concentration range 5 × 10^−5^ to 1 × 10^−2^ mol dm^−3^) to obtain the desired concentrations of both dye and adsorbent in the final mixture. The mixtures were placed in an orbital shaker (SK-O180-S, DLAB Scientific Co., Ltd., Beijing, China) at 170 rpm for a predetermined contact time. After that, the mixtures were centrifuged using a MiniSpin^®^ microcentrifuge (Eppendorf AG, Hamburg, Germany) (14,500 rpm, 1 min) to separate the adsorbent particles from the liquid phase. Supernatants were then collected and analyzed by UV-Vis spectrophotometry (PerkinElmer Inc., Waltham, MA, USA). The absorption spectra were recorded, and changes in absorbance at each dye’s characteristic maximum absorption wavelength (λ_max_) were monitored to determine the residual dye concentration in solution. Control experiments were performed under identical conditions without an adsorbent. All adsorption experiments were performed in triplicate, and the results are presented as mean ± standard deviation. Statistical analysis of the effects of pH, temperature, adsorbent dosage, and contact time was performed using one-way analysis of variance (ANOVA), followed by Tukey’s HSD post hoc test. Statistical analyses were carried out using Statistica software (Version 8.0, TIBCO Software Inc., Palo Alto, CA, USA). Different lowercase letters indicate statistically significant differences among mean values within the same adsorbent–dye system, whereas identical letters indicate no statistically significant difference. The experimental adsorption data obtained under these conditions were further analyzed using kinetic models, including the pseudo-first-order (PFO), pseudo-second-order (PSO), Elovich, and intraparticle diffusion models, as well as equilibrium isotherm models according to Freundlich, Langmuir, Temkin, and Dubinin-Radushkevich. The detailed description of the applied kinetic and equilibrium models, including equations, parameter definitions, and original references, is provided in the Supplementary Materials [54,55,56,57,58,59,60,61,62,63,64,65].

Dynamic adsorption performance, regeneration ability, and reusability of the selected materials were investigated using nylon syringe filters (0.22 μm pore size) loaded with 5 mg of each adsorbent. A volume of 1 mL of a dye solution at a concentration of 5 × 10^−5^ mol dm^−3^ was passed through the filters using a disposable 2 mL Luer Slip syringe (Nipro, Shanghai, China) under gentle manual pressure, and the absorbance of the effluent was measured to determine the dye removal efficiency. Regeneration was performed by washing the filters with 1 mL of 50% (*v*/*v*) ethanol under the same conditions, followed by a new adsorption cycle using fresh dye solution. This procedure was repeated over five consecutive cycles. In addition, UV-Vis spectra of the eluents were recorded to assess the potential for dye recovery and reuse.

### 3.3. Molecular Dimension Calculations

Three-dimensional molecular structures of dyes were obtained as Structure Data Files from the PubChem database. The molecular structures were processed using a custom Python (Version 3.11) script to estimate their molecular dimensions. Briefly, the atomic coordinates were aligned along the principal molecular axes using principal component analysis, after which the minimum three-dimensional bounding box was determined. Molecular dimensions were calculated from the minimum and maximum atomic coordinates along each principal axis. In addition, approximate van der Waals dimensions were estimated by extending the molecular boundaries according to the van der Waals radii of the constituent atoms. The calculated dimensions are reported as length × width × thickness (nm) and were used solely for qualitative comparison with the characteristic pore widths obtained by NLDFT analysis. Since the calculations were performed on isolated molecular conformers, the obtained values should not be interpreted as rigid kinetic diameters in aqueous solution.

## 4. Conclusions

Non-activated porous carbon materials were successfully prepared from *Juglans regia* and *Allium sativum* biomass through simple pyrolysis, providing a sustainable route for converting renewable biomass into value-added adsorbents without chemical activation. The designation “zero-waste conversion” is supported by the complete utilization of the biomass feedstocks as carbon precursors, the absence of chemical activating agents, the minimization of secondary waste generation during synthesis, and the recovery of the adsorbed dyes during regeneration, enabling both adsorbent reuse and dye recovery.

Carbonization at 900 °C dramatically improved the textural properties of both materials, yielding a specific surface area of 790 m^2^ g^−1^ for JR900 and 177 m^2^ g^−1^ for AS900, with predominantly microporous structures (1.18 and 1.64 nm, respectively). The adsorption performance of JR900 and AS900 is governed by the interplay of microporous structure, surface charge, and structural heterogeneity, resulting in energetically diverse adsorption sites. Kinetic analysis indicated that the pseudo-second-order model provided the best description of the adsorption process, while intraparticle diffusion analysis revealed a multistep mechanism involving external mass transfer and pore diffusion. Equilibrium data were well described by both Langmuir and Freundlich isotherms, indicating the coexistence of monolayer adsorption and surface heterogeneity.

JR900 exhibited the highest adsorption capacities for MB (321 mg g^−1^) and RB (304 mg g^−1^), whereas AS900 showed the highest capacity for MG (278 mg g^−1^), demonstrating that adsorption performance depends not only on surface area but also on pore accessibility and dye molecular structure. Regeneration studies demonstrated stable dynamic adsorption of RB (approximately 100% removal over five consecutive cycles for JR900), nearly complete dye recovery during desorption, and excellent adsorbent reusability, while the lower dynamic performance of CV and MG was primarily associated with slower diffusion and limited contact time. Overall, these findings demonstrate that non-activated biomass-derived carbons can achieve competitive adsorption performance without chemical activation while simultaneously enabling adsorbent regeneration and dye recovery, making them promising materials for sustainable and circular water treatment technologies.

## Figures and Tables

**Table 1:** Kinetic parameters for dye (5 × 10^−5^ mol dm^−3^) adsorption onto JR900 and AS900.

Material	JR900				AS900			
Dye	MB	RB	CV	MG	MB	RB	CV	MG
Pseudo-first-order kinetic model								
q_e_ [mg g^−1^]	15.5 ± 0.3	22 ± 2	14.7 ± 0.9	23 ± 2	15.9 ± 0.1	23.0 ± 0.1	12 ± 2	21 ± 4
k_1_ [min^−1^]	1.00 ± 0.04	1.01 ± 0.7	0.10 ± 0.01	0.25 ± 0.03	0.751 ± 0.003	0.740 ± 0.003	0.52 ± 0.08	0.58 ± 0.09
χ^2^	0.611	8.487	5.880	15.935	0.091	6.319	6.632	16.999
R^2^	0.979	0.876	0.824	0.810	0.997	0.915	0.751	0.766
Pseudo-second-order kinetic model								
q_e_ [mg g^−1^]	15.98 ± 0.03	23.7 ± 0.4	15.7 ± 0.6	24.5 ± 0.6	16.4 ± 0.1	24.1 ± 0.3	13.9 ± 0.8	22.8 ± 0.9
k_2_ ×10^2^ [mg min^−1^ g^−1^]	8.81 ± 0.01	5.23 ± 0.04	1.04 ± 0.03	1.47 ± 0.03	6.66 ± 0.01	3.82 ± 0.03	3.6 ± 0.2	2.4 ± 0.2
χ^2^	0.109	4.297	3.271	8.194	0.260	2.693	3.754	9.792
R^2^	0.996	0.937	0.902	0.902	0.992	0.964	0.859	0.865
Elovich kinetic model								
α [mg g^−1^ min^−1^]	7900 ± 300	5100 ± 300	23.3 ± 0.6	92 ± 2	3000 ± 500	1500 ± 200	86 ± 2	136 ± 7
β [g mg^−1^]	0.87 ± 0.03	0.55 ± 0.02	0.520 ± 0.003	0.355 ± 0.006	0.79 ± 0.05	0.49 ± 0.04	0.652 ± 0.004	0.394 ± 0.004
χ^2^	3.133	7.761	0.317	1.981	5.735	10.274	0.598	1.521
R^2^	0.895	0.886	0.991	0.976	0.830	0.862	0.978	0.979
Intraparticle diffusion model								
I part								
C [mg g^−1^]	1.59	0.21	0.04	−0.15	0.64	−0.34	0.15	−0.07
k_id_ [mg g^−1^ min^−0.5^]	5.97	13.95	5.13	10.25	6.61	12.89	6.42	11.02
R^2^	0.874	0.985	0.996	0.984	0.980	0.952	0.963	0.997
II part								
C [mg g^−1^]	12.16	8.88	6.18	12.21	13.50	8.86	6.94	11.87
k_id_ [mg g^−1^ min^−0.5^]	0.68	3.68	0.72	1.05	0.64	3.75	0.64	1.00
R^2^	0.920	0.815	0.861	0.844	--	0.908	0.903	0.932
III part								
C [mg g^−1^]	15.99	23.95	14.64	25.86	15.993	23.95	15.53	25.87
k_id_ [mg g^−1^ min^−0.5^]	0	0	0.088	0.054	0	0	0.013	0.021
R^2^	1	--	--	--	1	--	--	--

**Table 2:** Isotherm parameters for dye adsorption onto JR900 and AS900 at 25 °C.

Material	JR900				AS900			
Dye	MB	RB	CV	MG	MB	RB	CV	MG
Freundlich isotherm model								
K_F_ [(dm^3^ mg^−1^)^1/n^]	17.2 ± 0.3	52.1 ± 0.7	18.1 ± 0.9	19.0 ± 0.7	17.2 ± 0.4	75 ± 3	13.7 ± 0.7	24.9 ± 0.8
n	2.76 ± 0.05	4.71 ± 0.06	10.5 ± 0.5	3.57 ± 0.06	3.16 ± 0.05	7.9 ± 0.2	5.1 ± 0.2	3.7 ± 0.2
χ^2^	737	844	10	697	393	809	49	943
R^2^	0.949	0.936	0.862	0.906	0.941	0.873	0.892	0.907
Langmuir isotherm model								
K_L_ × 10^3^ [dm^3^ mg^−1^]	3.39 ± 0.02	5.85 ± 0.05	200 ± 30	1.76 ± 0.04	4.71 ± 0.03	12.4 ± 0.7	9 ± 1	2.24 ± 0.06
q_max_ [mg g^−1^]	321 ± 4	304 ± 7	35 ± 3	243 ± 6	217 ± 4	210 ± 10	65 ± 9	278 ± 8
χ^2^	233	1029	11	619	161	738	124	531
R^2^	0.984	0.922	0.847	0.916	0.976	0.884	0.724	0.948
Temkin isotherm model								
K_T_ [dm^3^ mg^−1^]	0.0752 ± 0.0001	0.171 ± 0.005	168 ± 8	0.12 ± 0.01	0.102 ± 0.005	3.3 ± 0.2	1.8 ± 0.1	0.133 ± 0.006
b_T_ [J g mol^−1^ mg^−1^]	46.1 ± 0.4	53.8 ± 0.8	838 ± 9	78 ± 2	68.3 ± 0.5	110 ± 20	340 ± 30	66.4 ± 0.9
χ^2^	577	810	5	942	192	784	61	877
R^2^	0.960	0.939	0.923	0.873	0.971	0.877	0.863	0.913
Dubinin-Radushkevich isotherm model								
q_DR_ [mg g^−1^]	280 ± 40	260 ± 40	30 ± 10	200 ± 100	180 ± 30	200 ± 30	50 ± 20	200 ± 60
K_DR_ × 10^3^ [mol^2^ J^−2^]	6.0 ± 0.2	1.31 ± 0.1	0.0016 ± 0.0006	0.6 ± 0.1	1.4 ± 0.1	1.1 ± 0.1	0.002 ± 0.001	0.6 ± 0.2
E [J mol^−1^]	9.2 ± 0.7	19 ± 4	600 ± 200	30 ± 10	20 ± 4	22 ± 4	500 ± 200	30 ± 10
χ^2^	1876	2488	30	3868	1118	853	374	4252
R^2^	0.871	0.812	0.576	0.477	0.833	0.866	0.166	0.580

**Table 3:** Calculated dimensions of the investigated dye cations and comparison with the characteristic pore widths of JR900 and AS900 obtained using the NLDFT method.

Dye	Molecular Structure and Geometry	Geometric Dimensions from Atomic Centers * L × W × T [nm]	Approximate van der Waals Dimensions ** L × W × T [nm]	Implications for Pore Accessibility (JR900–1.18 nm; AS900–1.64 nm) ***
MB	Nearly planar phenothiazine structure	1.410 × 0.566 × 0.299	1.650 × 0.806 × 0.539	Small effective cross-section and planar geometry favor access to narrow pores
RB	Xanthene chromophore with a rotatable carboxyphenyl group; conformationally adaptable	1.496 × 1.151 × 0.681	1.736 × 1.391 × 0.921	Large overall envelope, but orientation, conformational freedom, and adsorption at wider pores or external sites may facilitate uptake
CV	Highly branched and relatively symmetric triphenylmethane structure with three dimethylamino groups	1.389 × 1.157 × 0.439	1.629 × 1.397 × 0.679	Large effective cross-section and propeller-like geometry impose strong steric and orientational restrictions
MG	Triphenylmethane structure with two dimethylamino groups; less sterically hindered than CV	1.357 × 1.044 × 0.442	1.597 × 1.288 × 0.682	Slightly smaller effective cross-section than CV may permit access to a larger fraction of the pore network

* Geometric dimensions were calculated from the three-dimensional SDF coordinates using a principal-component-aligned bounding box. ** Approximate van der Waals dimensions were estimated by including the van der Waals radii of the constituent atoms. These values describe isolated molecular conformers and should not be interpreted as rigid kinetic diameters in aqueous solution. *** The NLDFT values represent characteristic pore widths within a distribution of pore sizes rather than uniform cylindrical pore diameters.

**Table 4:** Summary of the experimental conditions used for batch adsorption studies.

	Adsorption Parameters				
	Material Concentration [mg mL^−1^]	Adsorption Temperature [°C]	pH	Contact Time [min]	Dye Concentration [mol dm^−3^]
Effect of material concentration	0.2–5.0	25	7.0	60	5 × 10^−5^
Effect of temperature	1.0	25–40	7.0	60	5 × 10^−5^
Effect of pH	1.0	25	3.0–11.0	60	5 × 10^−5^
Kinetic studies	1.0	25	7.0	0–1440	5 × 10^−5^
Equilibrium (isotherm) studies	1.0	25	7.0	1440	5 × 10^−5^–1 × 10^−2^

## Data Availability

Data will be made available on request.

## Supplementary Materials

The following supporting information can be downloaded at: https://www.mdpi.com/article/10.3390/molecules31152678/s1, Table S1. Effect of adsorbent dosage on the removal efficiency (%) of MB, RB, CV, and MG using JR400, JR900, AS400, and AS900. Table S2. Effect of pH on the removal efficiency (%) of MB, RB, CV, and MG using JR400, JR900, AS400, and AS900. Table S3. Effect of temperature on the removal efficiency (%) of MB, RB, CV, and MG using JR400, JR900, AS400, and AS900. Table S4. Effect of contact time on the adsorption capacity qt [mg g^−1^] of MB, RB, CV, and MG onto JR900 and AS900. Table S5. Adsorption and desorption efficiency (%) of MB, RB, CV, and MG onto JR900 and AS900. Table S6. Comparative study with other biomass based carbon materials for the adsorption of investigated cationic dyes avalivable in literature. Figure S1. FTIR spectra of the adsorbents prior and after 5th cycle of adsorption and desorption.

## References

[1] Lellis B., Fávaro-Polonio C.Z., Pamphile J.A., Polonio J.C. (2019). Effects of textile dyes on health and the environment and bioremediation potential of living organisms. Biotechnol. Res. Innov..

[2] Oladoye P.O., Ajiboye T.O., Omotola E.O., Oyewola O.J. (2022). Methylene blue dye: Toxicity and potential elimination technology from wastewater. Results Eng..

[3] Jain R., Sharma N., Bhargava M. (2003). Electrochemical degradation of rhodamine B dye in textile and paper industries effluent. J. Sci. Ind. Res..

[4] Lazar M.M., Damaschin R.P., Volf I., Dinu M.V. (2024). Deep Cleaning of Crystal Violet and Methylene Blue Dyes from Aqueous Solution by Dextran-Based Cryogel Adsorbents. Gels.

[5] Sharma J., Sharma S., Soni V. (2023). Toxicity of malachite green on plants and its phytoremediation: A review. Reg. Stud. Mar. Sci..

[6] Kumar M., Mishra A., Patel S.K., Kushwaha J., Singh S., Mishra V., Singh D., Singh V., Giri B.S., Singhania R.R. (2025). Environmental Impacts and Strategies for Bioremediation of Dye-Containing Wastewater. Bioengineering.

[7] Cui M.-H., Cui D., Gao L., Wang A.-J., Cheng H.-Y. (2016). Azo dye decolorization in an up-flow bioelectrochemical reactor with domestic wastewater as a cost-effective yet highly efficient electron donor source. Water Res..

[8] https://sdgs.un.org/goals.

[9] Collivignarelli M.C., Abbà A., Carnevale Miino M., Damiani S. (2019). Treatments for color removal from wastewater: State of the art. J. Environ. Manag..

[10] Kuppusamy B., Mohamed Ismail F.R., Balakrishnan P., Kim S.C., Asrafali S.P., Periyasamy T. (2026). From Biomass to Adsorbent: A Comprehensive Review on Bio-Derived Carbons for Dye Removal. Polymers.

[11] Al-Tohamy R., Ali S.S., Li F., Okasha K.M., Mahmoud Y.A.G., Elsamahy T., Jiao H., Fu Y., Sun J. (2022). A critical review on the treatment of dye-containing wastewater: Ecotoxicological and health concerns of textile dyes and possible remediation approaches for environmental safety. Ecotoxicol. Environ. Saf..

[12] Hamad H.N., Idrus S. (2022). Recent Developments in the Application of Bio-Waste-Derived Adsorbents for the Removal of Methylene Blue from Wastewater: A Review. Polymers.

[13] Santoso E., Ediati R., Kusumawati Y., Bahruji H., Sulistiono D.O., Prasetyoko D. (2020). Review on recent advances of carbon based adsorbent for methylene blue removal from waste water. Mater. Today Chem..

[14] Varghese S., Ralph P., Kuzhiumparambil U. (2025). Sustainable carbon-rich materials from algae and biomass: Synthesis, characterisation, and applications. Results Eng..

[15] Cao S., Zhu R., Wu D., Su H., Liu Z., Chen Z. (2024). How hydrogen bonding and π-π interactions synergistically facilitate mephedrone adsorption by bio-sorbent: An in-depth microscopic scale interpretation. Environ. Pollut..

[16] Satyam S., Patra S. (2024). Innovations and challenges in adsorption-based wastewater remediation: A comprehensive review. Heliyon.

[17] Milanković V., Tasić T., Brković S., Potkonjak N., Unterweger C., Pašti I., Lazarević-Pašti T. (2024). The adsorption of chlorpyrifos and malathion under environmentally relevant conditions using biowaste carbon materials. J. Hazard. Mater..

[18] Amiry R.M., Mwankuna C.J., Mariki E.E., Mkoma S.L. (2025). Optimization and characterization of high surface area mesoporous activated carbon produced from *Syzygium cumini* leaves bio-waste by zinc chloride activation. Next Res..

[19] Saleem J., Moghal Z.K.B., Tahir F., Al-Ansari T., McKay G. (2025). Environmental Impacts and Adsorption Isotherms of Coconut Shell Activated Carbon: Effect of Acid Activation, Water, and Fuel. C.

[20] Ighalo J.O., Omoarukhe F.O., Ojukwu V.E., Iwuozor K.O., Igwegbe C.A. (2022). Cost of adsorbent preparation and usage in wastewater treatment: A review. Clean. Chem. Eng..

[21] Streit A.F.M., Pereira H.A., Moreno-Pérez J., Mendoza-Castillo D.I., Reynel-Ávila H.E., da Boit Martinello K., Silva L.F.O., Ahmad N., Mohandoss S., Bonilla-Petriciolet A. (2024). New study of the adsorption mechanism of different dye molecules by high porous sludge activated carbon produced from a dairy-treatment effluent plant. J. Environ. Chem. Eng..

[22] Molla A., Khatun N., Kim D. (2026). Biomass-derived carbon materials as adsorbent materials for CO_2_, organic dyes, antibiotics, and heavy metals—A review. Biomass Futures.

[23] Ania C., Bandosz T.J., Cazorla-Amorós D., Pereira M.F.R. (2026). Advancing Porous Carbons: Understanding the Importance of Surface Chemistry for the Energy–Environment Nexus. Chem. Rev..

[24] Chamorro F., Carpena M., Lourenço-Lopes C., Taofiq O., Otero P., Cao H., Xiao J., Simal-Gandara J., Prieto M.A. (2022). By-Products of Walnut (*Juglans regia*) as a Source of Bioactive Compounds for the Formulation of Nutraceuticals and Functional Foods. Biol. Life Sci. Forum.

[25] Rovera C., Carullo D., Bellesia T., Büyüktaş D., Ghaani M., Caneva E., Farris S. (2023). Extraction of high-quality grade cellulose and cellulose nanocrystals from different lignocellulosic agri-food wastes. Front. Sustain. Food Syst..

[26] Ge L., Zhao C., Chen S., Li Q., Zhou T., Jiang H., Li X., Wang Y., Xu C. (2022). An analysis of the carbonization process and volatile-release characteristics of coal-based activated carbon. Energy.

[27] Li C., Gong Y., Tian X., Yao J., Wang X., Niu B., Li G., Long D. (2026). From Organic Network to Graphitic Structure: Atomistic Insights into Carbonization Mechanisms of Representative Carbonaceous Precursors. J. Phys. Chem. C.

[28] Serrano-Lotina A., Portela R., Baeza P., Alcolea-Rodriguez V., Villarroel M., Ávila P. (2023). Zeta potential as a tool for functional materials development. Catal. Today.

[29] Sharma S.K., Sharma G., Sharma A., Bhardwaj K., Preeti K., Singh K., Kumar A., Pal V.K., Choi E.H., Singh S.P. (2022). Synthesis of silica and carbon-based nanomaterials from rice husk ash by ambient fiery and furnace sweltering using a chemical method. Appl. Surf. Sci. Adv..

[30] Basta A.H., Lotfy V.F. (2025). Optimizing the lignin nanoparticles from different pulping by-products in developing cotton-based nanocrystalline cellulose for UV-light blocking. Sci. Rep..

[31] Atta A., Abdeltwab E., Negm H., Al-Harbi N., Rabia M., Abdelhamied M.M. (2023). Fabrication of Polypyrrole/Graphene Oxide Polymer Nanocomposites and Evaluation of Their Optical Behavior for Optoelectronic Applications. J. Inorg. Organomet. Polym. Mater..

[32] Hernández-Rentero C., Marangon V., Olivares-Marín M., Gómez-Serrano V., Caballero Á., Morales J., Hassoun J. (2020). Alternative lithium-ion battery using biomass-derived carbons as environmentally sustainable anode. J. Colloid Interface Sci..

[33] Wang H., Li X., Peng J., Cai Y., Jiang J., Li Q. (2021). Control of the interface graphitized/amorphous carbon of biomass-derived carbon microspheres for symmetric supercapacitors. Nanoscale Adv..

[34] Sahadat Hossain M., Jahan S.A., Ahmed S. (2023). Crystallographic characterization of bio-waste material originated CaCO_3_, green-synthesized CaO and Ca(OH)_2_. Results Chem..

[35] Wei F., Jin S., Yao C., Wang T., Zhu S., Ma Y., Qiao H., Shan L., Wang R., Lian X. (2023). Revealing the Combined Effect of Active Sites and Intra-Particle Diffusion on Adsorption Mechanism of Methylene Blue on Activated Red-Pulp Pomelo Peel Biochar. Molecules.

[36] Ahmed M.J., Hameed B.H. (2019). Insights into the isotherm and kinetic models for the coadsorption of pharmaceuticals in the absence and presence of metal ions: A review. J. Environ. Manag..

[37] Paluch D., Bazan-Wozniak A., Nosal-Wiercińska A., Pietrzak R. (2026). Kinetic and Thermodynamic Studies of Methylene Blue Adsorption on Biomass-Derived Biocarbon Materials. Int. J. Mol. Sci..

[38] Yu X., Wei C., Wu H. (2015). Effect of molecular structure on the adsorption behavior of cationic dyes onto natural vermiculite. Sep. Purif. Technol..

[39] Milanković V., Tasić T., Brković S., Potkonjak N., Unterweger C., Pašti I., Lazarević-Pašti T. (2025). Sustainable carbon materials from biowaste for the removal of organophosphorus pesticides, dyes, and antibiotics. J. Environ. Manag..

[40] Sangsuk S., Napanya P., Tasen S., Baiya P., Buathong C., Keeratisoontornwat K., Suebsiri S. (2023). Production of non-activated biochar based on *Biden pilosa* and its application in removing methylene blue from aqueous solutions. Heliyon.

[41] Jiang D., Li H., Cheng X., Ling Q., Chen H., Barati B., Yao Q., Abomohra A., Hu X., Bartocci P. (2023). A mechanism study of methylene blue adsorption on seaweed biomass derived carbon: From macroscopic to microscopic scale. Process Saf. Environ. Prot..

[42] Li P., Zhao T., Zhao Z., Tang H., Feng W., Zhang Z. (2023). Biochar Derived from Chinese Herb Medicine Residues for Rhodamine B Dye Adsorption. ACS Omega.

[43] Kumbhar P., Patil S., Narale D., Sartape A., Jambhale C., Kim J.-H., Kolekar S. (2024). Biobased carbon for effective removal of rhodamine B and Cr(VI) from aqueous solution: Kinetic, isotherm and thermodynamic study. Biomass Convers. Biorefinery.

[44] Raji Y., Nadi A., Mechnou I., Saadouni M., Cherkaoui O., Zyade S. (2023). High adsorption capacities of crystal violet dye by low-cost activated carbon prepared from Moroccan Moringa oleifera wastes: Characterization, adsorption and mechanism study. Diam. Relat. Mater..

[45] Jabar J.M., Adebayo M.A., Owokotomo I.A., Odusote Y.A., Yılmaz M. (2022). Synthesis of high surface area mesoporous ZnCl_2_–activated cocoa (*Theobroma cacao* L.) leaves biochar derived *via* pyrolysis for crystal violet dye removal. Heliyon.

[46] Mosoarca G., Vancea C., Popa S., Dan M., Boran S. (2022). Crystal Violet Adsorption on Eco-Friendly Lignocellulosic Material Obtained from Motherwort (*Leonurus cardiaca* L.) Biomass. Polymers.

[47] Giri B.S., Sonwani R.K., Varjani S., Chaurasia D., Varadavenkatesan T., Chaturvedi P., Yadav S., Katiyar V., Singh R.S., Pandey A. (2022). Highly efficient bio-adsorption of Malachite green using Chinese Fan-Palm Biochar (*Livistona chinensis*). Chemosphere.

[48] Abewaa M., Mengistu A., Takele T., Fito J., Nkambule T. (2023). Adsorptive removal of malachite green dye from aqueous solution using Rumex abyssinicus derived activated carbon. Sci. Rep..

[49] Ahmad T., Manzar M.S., Firmansyah M.L., Khan S., Ahmed I.U., Younas M., Ullah N. (2026). Biomass-derived activated carbons from Ziziphus jujube and *Dodonaea viscosa* for the efficient removal of malachite green: A comparative adsorption performance and mechanisms. Int. J. Environ. Anal. Chem..

[50] Heidarinejad Z., Dehghani M.H., Heidari M., Javedan G., Ali I., Sillanpää M. (2020). Methods for preparation and activation of activated carbon: A review. Environ. Chem. Lett..

[51] Igliński B., Kujawski W., Kiełkowska U. (2023). Pyrolysis of Waste Biomass: Technical and Process Achievements, and Future Development—A Review. Energies.

[52] Garg S., Ali A., Ahmed S., Rumjit N.P., Ahmad S., Beig S.U.R. (2026). Cost-effective adsorbents for dye remediation: Recent breakthroughs and future prospects. Environ. Eng. Res..

[53] https://www.icdd.com.

[54] Krstić V., Bhanvase B., Sonawane S., Pawade V., Pandit A. (2021). Chapter 14—Role of zeolite adsorbent in water treatment. Handbook of Nanomaterials for Wastewater Treatment.

[55] Saleh T.A., Saleh T.A. (2022). Chapter 3–Kinetic models and thermodynamics of adsorption processes: Classification. Interface Science and Technology.

[56] Lagergren S. (1898). About the Theory of So-Called Adsorption of Soluble Substances. https://www.sid.ir/paper/563615/en.

[57] Ho Y.S., McKay G. (1999). Pseudo-second order model for sorption processes. Process Biochem..

[58] Roginsky S., Zeldovich Y.B. (1934). The catalytic oxidation of carbon monoxide on manganese dioxide. Acta Phys. Chem. USSR.

[59] Weber W.J., Morris J.C. (1963). Kinetics of adsorption on carbon from solution. J. Sanit. Eng. Div..

[60] Al-Ghouti M.A., Da’ana D.A. (2020). Guidelines for the use and interpretation of adsorption isotherm models: A review. J. Hazard. Mater..

[61] Freundlich H.M.F. (1906). Over the adsorption in solution. J. Phys. Chem..

[62] Langmuir I. (1918). The adsorption of gases on plane surfaces of glass, mica and platinum. J. Am. Chem. Soc..

[63] Temkin M. (1940). Kinetics of ammonia synthesis on promoted iron catalysts. Acta Physiochim URSS.

[64] Wang J., Guo X. (2020). Adsorption isotherm models: Classification, physical meaning, application and solving method. Chemosphere.

[65] Dubinin M. (1947). The equation of the characteristic curve of activated charcoal. Dokl. Akad. Nauk. SSSR.

