# Supplementary material for: Zero-Waste Conversion of Juglans regia and Allium sativum Biomass into Porous Carbons for Dye Removal and Recovery from Water
Source: Molecules. 2026 Jul 31;31(15):2678. doi: 10.3390/molecules31152678 (PMC13468325; doi:10.3390/molecules31152678)
Supplement: Supplementary file 1 [file molecules-31-02678-s001.zip › molecules-4442036-supplementary.pdf]

Supplementary Material

Zero-waste conversion of Juglans regia and Allium sativum biomass into porous carbons for dye removal and recovery from water

Nevena Radivojević [1], Tamara Terzić [1], Tamara Lazarević-Pašti [1], Igor Pašti [2,3], Nebojša Potkonjak [1], Aleksa Luković [1], Jasmina Mušović [1] and Vedran Milanković [1,*]

Adsorption and desorption efficiency

The adsorption performance was evaluated in terms of removal efficiency, equilibrium concentration (c_e), adsorbed concentration (c_a), and adsorption capacity (q_e), calculated according to the following expressions:

$$\text{Removal efficiency [\%]} = (1 - \frac{A}{A_0}) \times 100\% \quad (1)$$

$$c_e = \frac{A}{A_0} \times c_0 \quad (2)$$

$$c_a = c_0 - c_e \quad (3)$$

$$q_e = \frac{c_a \times M}{\gamma} \quad (4)$$

where A_0 represents the absorbance of the dye solution at λ_{max} in the absence of adsorbent, while A is the absorbance after adsorption. c_0, c_e, and c_a correspond to the initial, equilibrium, and adsorbed dye concentrations (mol dm^{-3}), respectively. M denotes the molar mass of the dye (mg mol^{-1}), and γ is the adsorbent concentration (g dm^{-3}).

For the regeneration experiments, the concentration of desorbed dye was determined from the absorbance of the desorption solution according to:

$$c_d = \frac{A_d}{A_0} \times c_0 \quad (5)$$

where A_d is the absorbance of the desorption solution measured at the characteristic wavelength after regeneration, and c_d is the concentration of desorbed dye (mol dm^{-3}).

The desorption efficiency was calculated as:

$$\text{Desorption efficiency [\%]} = \frac{c_d}{c_a} \times 100\%$$

where c_a is the amount of dye adsorbed during the preceding adsorption cycle.

Adsorption kinetics

Adsorption in porous adsorbents takes time to reach equilibrium due to mass transfer resistance. The process involves: external mass transfer across the boundary layer or diffusion film between the liquid phase and the adsorbent's surface; diffusion within the adsorbent particles, allowing the adsorbate to enter the pores; and the formation of physical or chemical bonds at the active sites within the adsorbent's pores. The slowest step dictates the adsorption rate, which may depend on either film or intraparticle diffusion [1].

Kinetic adsorption curves (q_t=f(t)) are analyzed to monitor adsorbate concentration changes over time, helping to identify the controlling kinetic mechanism. Models such as the pseudo-first-order, pseudo-second-order kinetics, Elovich, and intraparticle diffusion model are often used to describe the adsorption kinetics of organic compounds [2].

The Lagergren pseudo-first-order (PFO) model assumes that the rate of adsorbate uptake over time is directly proportional to the difference between the saturation concentration and the amount of adsorbate adsorbed over time. This model is generally applicable during the initial stage of the adsorption process. It is often observed that adsorption kinetics follow this PFO equation when the process involves diffusion through the interface. The equation representing this model is:

$$q_t = q_e(1 - e^{-k_1 t}) \tag{6}$$

, where q_t is the amount of adsorbate at the time (mg g^{-1}), q_e is the equilibrium adsorption capacity (mg g^{-1}), and k_1 is the rate constant of PFO (min^{-1}) [3].

The pseudo-second-order (PSO) kinetic model extends this analysis to systems involving the assumption that the adsorption rate is dependent on concentrations of both adsorbate and adsorbent. The equation representing this model is:

$$q_t = \frac{q_e^2 k_2 t}{1 + q_e k_2 t} \tag{7}$$

, where k_2 is the adsorption rate of PSO (g mg^{-1} min^{-1}) [4].

The Elovich kinetic model is an empirical model frequently used to analyze adsorption kinetics and effectively describes second-order kinetics based on the assumption that the surface has energetic heterogeneity. The equation representing this model is:

$$q_t = \frac{1}{\beta}(1 + \alpha\beta t) \tag{8}$$

, where α is the initial adsorption rate (mg g^{-1} min^{-1}), and β is the desorption constant (g mg^{-1}) [5].

The intraparticle diffusion (IPD) kinetic model is fundamental for understanding the adsorption process within porous materials. It provides insights into how adsorbate molecules diffuse into the interior of adsorbent particles and interact with the surface sites. By considering diffusion mechanisms such as pore diffusion and surface diffusion, this model clarifies the rates at which adsorption occurs within the particle. The equation representing this model is:

$$q_t = k_{id} t^{0.5} + C \tag{9}$$

, where k_{id} is the adsorption rate constant of the IPD model (mg g^{-1} $min^{-0.5}$), C is a boundary layer (mg g^{-1}) [2, 6].

Adsorption isotherms

An adsorption isotherm (q_e=f(C_e); T=const.) represents the relationship between the concentration of adsorbate on the surface of the adsorbent (q_e (mg g^{-1})) and the equilibrium concentration of adsorbate in the solution (C_e (mg dm^{-3})) at a constant temperature and under given conditions.

Adsorption isotherms provide insights into the mechanisms of adsorption and help estimate adsorption capacity. Numerous theoretical and empirical isotherm models have been developed. The most frequently used

isotherm models for the description of adsorption are the Freundlich, Langmuir, Temkin, and Dubinin-Radushkevich isotherm models [7].

The Freundlich adsorption isotherm (Eq. 17) describes the relationship between the adsorbed amount of a substance and the equilibrium pressure or concentration of the adsorbate. The equation is used to model the relationship between adsorption and the available surface area of materials. Freundlich isotherm model states that adsorption increases exponentially with equilibrium concentration until the adsorption sites have all been saturated and the concentration of the adsorbent has been maximized. Freundlich's isotherm model predicts adsorption on an energetically heterogeneous surface, and adsorbed molecules interact with each other. If the value $n > 1$, adsorption is favored; if $n<1$, it is not favored; and if $n=1$, adsorption follows a linear function. K_F is the Freundlich adsorption coefficient ($(mg\ g^{-1})(dm^3\ g^{-1})^{1/n}$), representing the quantity of adsorbate adsorbed onto the adsorbent for a unit concentration of adsorbate in solution. It provides a measure of how well the adsorbent can adsorb the adsorbate at lower concentrations [7, 8].

$$q_e = K_F C_e^{\frac{1}{n}} \tag{10}$$

The Langmuir adsorption isotherm (Eq. 18) describes the relationship between the adsorbed amount of a substance and the equilibrium concentration of the adsorbate. This equation is used to model the adsorption process and to quantify the adsorption potential of a substance. The Langmuir isotherm states that adsorption increases linearly with equilibrium pressure until the adsorption sites have been saturated and the adsorption capacity of the material has been reached. Langumir's isotherm model proposes that adsorbate molecules are adsorbed on an energetically homogenous surface without the interaction between adsorbed molecules. It indicates that all active sites are energetically equivalent and that adsorption is reached when a monolayer is formed. K_L ($dm^3\ mg^{-1}$) value indicates the affinity of the adsorbate molecules towards the adsorbent, and q_{max} ($mg\ g^{-1}$) value implies the maximum adsorption capacity of the monolayer [7, 9].

$$q_e = \frac{q_{max} K_L C_e}{1 + K_L C_e} \tag{11}$$

The Temkin isotherm model (Eq. 19) assumes that the adsorption heat of all molecules decreases linearly with the increase in coverage of the adsorbent surface and that adsorption is characterized by a uniform distribution of binding energies up to a maximum binding energy. This isotherm model includes Temkin's isotherm constant b_T ($J\ g\ mol^{-1}\ mg^{-1}$) which is related to the adsorption energy of the system. The higher the adsorption energy, the higher the b_T value, and the more readily adsorbed the material is onto the surface of the solid. K_T ($dm^3\ mg^{-1}$) reflects the equilibrium binding constant, indicating the affinity of the adsorbate towards the adsorbent [7, 10].

$$q_e = \frac{RT}{b_T} \ln K_T C_e \tag{12}$$

The Dubinin-Radushkevich adsorption isotherm (Eq. 20) is an empirical adsorption isotherm model used to describe the relationship between adsorptive material and equilibrium adsorption pressure. It accounts for adsorption in both open and closed environments and for systems in which non-ideality of the adsorptive media is present. This isotherm gives the parameters q_{DR}, which is a theoretical isotherm saturation capacity ($mg\ g^{-1}$), and K_{DR}, which is the Dubinin-Radushkevich isotherm constant ($mol^2\ J^{-2}$). Polanyi potential ($\mathcal{E}=RT\ln(1+1/Ce)$) helps to describe the energy involved in the adsorption process [7].

$$q_e = q_{DR} e^{-K_{DR}\varepsilon^2} \tag{13}$$

Adsorption mean free energy E (J mol^{-1}) can be calculated from the Dubinin-Radushkevich adsorption isotherm model using the following equation:

$$E = \frac{1}{\sqrt{2K_{DR}}} \tag{14}$$

If the value of E > 8000 J mol^{-1}, the preferred adsorption mechanism is chemisorption, and if E< 8000 J mol^{-1}, physisorption is the probable process of adsorption [7, 11, 12].

Table S1. Effect of adsorbent dosage on the removal efficiency (%) of MB, RB, CV, and MG using JR400, JR900, AS400, and AS900.

	Removal efficiency [%]															
	JR400				JR900				AS400				AS900			
c_{mat} [mg mL^{-1}]	MB	RB	CV	MG	MB	RB	CV	MG	MB	RB	CV	MG	MB	RB	CV	MG
0.2	2.30±0.33^{a}	0.00±0.00^{a}	0.00±0.00^{a}	0.00±0.00^{a}	40.04±1.03^{c}	52.42±0.99^{d}	0.00±0.00^{d}	0.00±0.00^{b}	1.78±0.18^{a}	0.00±0.00^{a}	0.00±0.00^{a}	0.00±0.00^{a}	24.49±0.93^{b}	86.97±4.35^{b}	0.00±0.00^{b}	0.00±0.00^{a}
0.5	5.93±0.30^{b}	10.87±0.85^{b}	0.00±0.00^{a}	0.00±0.00^{a}	96.07±2.58^{a}	56.10±1.58^{c}	38.34±0.95^{c}	15.77±0.95^{c}	5.16±0.36^{b}	0.00±0.00^{a}	0.00±0.00^{a}	3.25±0.33^{b}	79.15±2.05^{c}	100.00±3.56^{a}	18.73±0.94^{c}	20.15±1.01^{b}
1.0	13.53±0.85^{c}	38.91±1.95^{c}	4.28±0.30^{a}	5.12±0.29^{a}	98.37±3.11^{b}	87.81±1.05^{d}	86.15±2.06^{d}	80.84±1.06^{d}	14.40±0.85^{c}	39.06±2.06^{b}	8.02±0.85^{b}	4.15±0.56^{b}	97.68±1.02^{a}	100.00±1.53^{a}	88.17±3.59^{d}	59.69±1.53^{c}
2.0	32.23±2.05^{d}	80.82±5.05^{d}	8.08±0.58^{b}	6.22±0.59^{a}	98.03±1.06^{ab}	100.00±3.58^{a}	100.00±3.59^{a}	100.00±3.85^{a}	23.18±2.08^{d}	62.38±3.12^{c}	8.45±0.85^{c}	6.15±0.31^{c}	97.82±2.40^{a}	100.00±2.53^{a}	98.71±2.06^{a}	99.33±3.59^{d}
5.0	73.93±5.05^{e}	100.00±2.06^{e}	36.91±2.06^{c}	9.15±0.62^{a}	98.05±2.84^{ab}	100.00±2.58^{a}	100.00±2.06^{a}	100.00±2.06^{a}	72.66±4.85^{e}	74.51±4.53^{d}	9.89±1.53^{d}	9.13±0.46^{d}	97.39±4.03^{a}	100.00±0.93^{a}	98.91±1.98^{a}	99.15±2.48^{d}

Values are presented as mean ± standard deviation (n = 3). Different lowercase letters within each column indicate statistically significant differences between experimental conditions according to one-way ANOVA followed by Tukey's HSD post hoc test. Identical letters indicate no statistically significant difference.

Table S2. Effect of pH on the removal efficiency (%) of MB, RB, CV, and MG using JR400, JR900, AS400, and AS900

	Removal efficiency [%]															
	JR400				JR900				AS400				AS900			
pH	MB	RB	CV	MG	MB	RB	CV	MG	MB	RB	CV	MG	MB	RB	CV	MG
pH	MB	RB	CV	MG	MB	RB	CV	MG	MB	RB	CV	MG	MB	RB	CV	MG
3	15.64±0.83^{d}	32.65±0.85^{a}	2.08±0.53^{c}	1.98±0.19^{c}	95.42±2.25^{a}	80.46±2.05^{a}	88.06±2.40^{a}	59.34±3.00^{b}	0.00±0.00^{a}	8.41±0.42^{b}	0.00±0.00^{a}	3.73±0.36^{a}	98.76±4.94^{a}	100.00±1.02^{a}	81.73±1.05^{b}	50.62±1.25^{b}
5	8.16± 0.76^{a}	25.54±0.58^{c}	3.07±0.36^{d}	5.29±0.35^{d}	97.75±3.52^{ab}	83.46±2.03^{b}	82.28±4.11^{c}	69.43±2.03^{c}	5.45±0.27^{b}	17.29±0.86^{a}	3.95±0.20^{c}	4.98±0.59^{b}	98.18±3.25^{a}	100.00±1.25^{a}	97.00±0.53^{a}	69.74±2.16^{c}
7	13.52±1.05^{c}	38.75±1.21^{d}	4.86±0.86^{e}	6.46±0.55^{e}	98.46±1.25^{ab}	87.75±1.25^{c}	88.22±1.03^{a}	75.96±1.26^{a}	14.40±0.72^{c}	22.90±1.14^{d}	8.46±0.42^{e}	3.58±0.13^{a}	95.85±2.25^{b}	100.00±2.25^{a}	89.88±2.03^{c}	81.75±3.01^{a}
9	4.91±0.25^{b}	33.46±0.98^{a}	1.46±0.07^{b}	6.89±0.52^{b}	100.00±3.52^{b}	98.75±0.98^{b}	95.82±0.99^{b}	77.77±4.53^{a}	18.75±0.94^{d}	17.75±0.89^{a}	6.48±0.56^{d}	6.06±0.58^{c}	96.90±1.54^{ab}	100.00±0.81^{a}	97.63±3.06^{a}	87.82±1.25^{d}

Values are presented as mean ± standard deviation (n = 3). Different lowercase letters within each column indicate statistically significant differences between experimental conditions according to one-way ANOVA followed by Tukey's HSD post hoc test. Identical letters indicate no statistically significant difference.

Table S3. Effect of temperature on the removal efficiency (%) of MB, RB, CV, and MG using JR400, JR900, AS400, and AS900.

	Removal efficiency [%]															
	JR400				JR900				AS400				AS900			
T [°C]	MB	RB	CV	MG	MB	RB	CV	MG	MB	RB	CV	MG	MB	RB	CV	MG
25	13.53±1.25^{b}	38.91±0.72^{a}	0.00±0.00^{a}	1.90±0.22^{a}	98.37±2.25^{b}	87.81±2.59^{b}	86.15±2.55^{b}	80.84±2.06^{a}	14.40±0.85^{a}	39.06±1.61^{a}	8.02±0.68^{b}	1.90±0.22^{a}	97.68±2.65^{a}	100.00±2.26^{a}	88.17±2.51^{b}	59.69±1.52^{a}
30	15.45±0.77^{c}	50.50±0.95^{b}	3.44±0.58^{b}	2.55±0.56^{a}	100.00±1.26^{a}	98.13±1.52^{c}	92.46±3.05^{c}	82.75±0.99^{b}	17.67±0.66^{b}	55.48±3.06^{b}	9.10±0.46^{c}	3.75±0.53^{b}	100.00±3.16^{a}	100.00±1.51^{a}	83.80±3.45^{a}	74.74±3.74^{b}
35	17.52±2.23^{a}	71.46±3.57^{c}	7.56±0.99^{c}	3.44±0.37^{b}	100.00±0.90^{a}	100.00±2.18^{a}	100.00±1.06^{a}	86.48±3.12^{c}	18.73±1.03^{c}	89.74±3.51^{c}	10.14±1.55^{a}	5.87±0.96^{c}	100.00±1.05^{a}	100.00±0.96^{a}	93.88±1.07^{c}	82.20±2.06^{c}
40	17.65±0.59^{a}	100.00±3.59^{d}	9.73±1.59^{d}	5.87±0.78^{c}	100.00±3.03^{a}	100.00±0.85^{a}	100.00±2.48^{a}	88.45±2.56^{d}	19.45±1.58^{d}	100.00±2.53^{d}	10.46±0.94^{a}	7.46±0.37^{d}	100.00±0.98^{a}	100.00±3.19^{a}	98.48±3.65^{d}	84.48±1.05^{d}

Values are presented as mean ± standard deviation (n = 3). Different lowercase letters within each column indicate statistically significant differences between experimental conditions according to one-way ANOVA followed by Tukey's HSD post hoc test. Identical letters indicate no statistically significant difference.

Table S4. Effect of contact time on the adsorption capacity q_t [mg g^{-1}] of MB, RB, CV, and MG onto JR900 and AS900.

	q_t [mg g^{-1}]							
	JR900				AS900			
t [min]	MB	RB	CV	MG	MB	RB	CV	MG
0	0.00±0.00^{b}	0.00±0.00^{b}	0.00±0.00^{a}	0.00±0.00^{d}	0.00±0.00^{b}	0.00±0.00^{b}	0.00±0.00^{b}	0.00±0.00^{c}
0.5	6.48±0.32^{c}	10.78±0.54^{c}	3.80±0.19^{a}	6.57±0.33^{e}	5.50±0.28^{c}	7.60±0.38^{c}	5.20±0.26^{c}	7.50±0.38^{d}
1	9.62±0.48^{d}	13.66±0.68^{d}	5.07±0.25^{a}	10.47±0.52^{f}	8.16±0.41^{d}	13.38±0.67^{d}	6.21±0.31^{d}	11.11±0.56^{e}
5	13.80±0.69^{e}	15.15±0.76^{e}	6.98±0.35^{ac}	13.88±0.69^{g}	14.94±0.75^{e}	15.87±0.79^{e}	8.15±0.41^{e}	13.41±0.67^{f}
15	14.52±0.56^{f}	23.95±1.20^{a}	9.95±0.50^{ac}	16.48±0.82^{h}	15.99±1.26^{a}	23.95±1.20^{a}	9.63±0.48^{f}	16.79±0.33^{g}
30	15.99±0.26^{a}	23.95±0.53^{a}	11.80±0.59^{bc}	21.16±1.06^{a}	15.99±0.80^{a}	23.95±0.85^{a}	11.36±0.57^{g}	19.15±0.79^{a}
60	15.99±0.35^{a}	23.95±0.79^{a}	12.75±0.64^{b}	22.44±0.89^{ab}	15.99±0.49^{a}	23.95±1.30^{a}	13.15±0.66^{h}	19.91±0.39^{a}
120	15.99±0.85^{a}	23.95±1.20^{a}	14.51±0.73^{bd}	23.56±0.76^{b}	15.99±0.55^{a}	23.95±0.75^{a}	14.25±0.71^{i}	23.18±0.47^{h}
240	15.99±0.12^{a}	23.95±0.52^{a}	15.98±0.80^{bd}	26.69±0.96^{c}	15.99±0.26^{a}	23.95±1.00^{a}	15.73±0.79^{a}	26.20±0.65^{b}
1440	15.99±0.86^{a}	23.95±0.85^{a}	17.93±0.33^{d}	27.89±0.32^{c}	15.99±0.80^{a}	23.95±1.20^{a}	16.02±0.80^{a}	26.68±0.25^{b}

Values are presented as mean ± standard deviation (n = 3). Different lowercase letters within each column indicate statistically significant differences between experimental conditions according to one-way ANOVA followed by Tukey's HSD post hoc test. Identical letters indicate no statistically significant difference.

Table S5. Adsorption and desorption efficiency (%) of MB, RB, CV, and MG onto JR900 and AS900

	Efficiency [%]															
	JR900								AS900							
	adsorption				desorption				adsorption				desorption			
Cycle	MB	RB	CV	MG	MB	RB	CV	MG	MB	RB	CV	MG	MB	RB	CV	MG
0	51.91±0.35^{b}	100.00±1.19^{a}	4.69±0.01^{a}	20.10±2.15^{b}	48.12±1.72^{b}	94.46±1.10^{a}	3.46±0.03^{a}	17.76±2.00^{a}	39.00±0.99^{b}	69.14±0.73^{b}	31..14±1.03^{b}	20.10±1.64^{a}	35.75±0.12^{b}	65.78±0.10^{b}	16.13±0.30^{a}	18.54±1.99^{a}
1	64.99±0.70^{c}	100.00±2.37^{a}	11.71±0.01^{b}	15.91±3.68^{ab}	46.26±3.49^{b}	97.46±2.05^{a}	8.45±0.03^{b}	14.16±1.16^{a}	38.42±2.04^{b}	73.86±1.50^{a}	15.00±1.15^{a}	23.73±0.73^{a}	35.15±0.34^{b}	70.12±0.21^{ab}	13.47±1.00^{ab}	20.50±1.12^{a}
2	26.92±3.06^{a}	100.00±3.91^{a}	3.16±0.20^{a}	15.24±1.11^{a}	24.46±4.16^{a}	96.48±0.97^{a}	2.56±0.41^{a}	14.99±0.78^{a}	35.76±1.86^{b}	74.07±0.35^{a}	17.50±0.50^{a}	24.48±1.78^{a}	30.45±0.51^{c}	71.56±0.18^{a}	16.22±2.05^{a}	22.10±1.42^{a}
3	27.54±0.32^{a}	100.00±0.43^{a}	3.32±0.01^{a}	16.19±0.30^{ab}	23.25±0.97^{a}	95.37±2.79^{a}	2.75±0.00^{a}	14.20±0.65^{a}	22.53±3.69^{a}	74.58±2.88^{a}	17.39±1.13^{a}	23.75±1.23^{a}	22.25±1.11^{a}	71.99±1.00^{a}	14.89±1.63^{ab}	22.00±2.10^{a}
4	28.51±1.68^{a}	100.00±4.02^{a}	1.50±0.01^{a}	15.51±0.50^{a}	25.25±0.62^{a}	98.76±1.98^{a}	1.25±0.00^{a}	13.48±0.69^{a}	26.53±1.50^{a}	74.62±1.78^{a}	15.49±0.99^{a}	23.13±2.05^{a}	23.46±0.05^{a}	71.35±1.62^{a}	12.64±0.01^{ab}	21.73±1.78^{a}
5	27.49±1.72^{a}	100.00±2.24^{a}	1.74±0.01^{a}	15.23±1.07^{a}	23.25±0.44^{a}	97.79±3.02^{a}	1.25±0.00a	13.96±0.09^{a}	23.16±0.89^{a}	74.22±1.43^{a}	13.91±0.40^{a}	23.47±1.89^{a}	21.05±0.92^{a}	70.85±0.03^{a}	11.50±0.50^{b}	21.00±1.52^{a}

Values are presented as mean ± standard deviation (n = 3). Different lowercase letters within each column indicate statistically significant differences between experimental conditions according to one-way ANOVA followed by Tukey's HSD post hoc test. Identical letters indicate no statistically significant difference.

Table S6. Comparative study with other biomass based carbon materials for the adsorption of investigated cationic dyes avalivable in literature

Dye	Precursor	T	Activation	q_{max} [mg g^{-1}]	Reference
MB	Spent coffee grounds	900	Chemical – KOH	8.2	[39]
			Chemical – H_3PO_4	1.23	
			Physical – CO_2	1.1	
			Chemical – KOH<br>Physical – CO_2	9.7	
	Biden pilosa	550	/	200	[40]
	Seaweed	500	Chemical - NaOH	243.839	[41]
	Juglans regia	900	/	321.62	This work
	Allium sativum	900	/	217.36	This work
RB	*Atropa belladonna L*	500	Chemical - $ZnCl_2$	263.19	[42]
			Chemical - H_3PO_4	309.11	
	Samanea saman	450	Chemical - 20% $ZnCl_2$	101.01	[43]
	Spent coffee grounds	900	Chemical – KOH	42.9	[39]
			Physical – CO_2	4.2	
			Chemical – KOH<br>Physical – CO_2	130	
	Juglans regia	900	/	304.32	This work
	Allium sativum	900	/	213.93	This work
CV	Cocoa leaves	700	/	190.7	[45]
			Chemical - $ZnCl_2$	253.3	
	Moringa oleifera seeds husks	900	Chemical - H_3PO_4	469.55	[44]
	Leonurus cardiaca L.	105	/	176.6	[46]
	Juglans regia	900	/	35.47	This work
	Allium sativum	900	/	65.38	This work
MG	*Livistona chinensis*	500	/	21.41	[47]
	Rumex abyssinicus	500	/	98.43	[48]
	Dodonaea viscosa	800	Chemical - $NaHCO_3$	208.05	[49]
	Ziziphus jujube	800	Chemical- $NaHCO_3$	226.3	
	Juglans regia	900	/	243.25	This work
	Allium sativum	900	/	277.60	This work

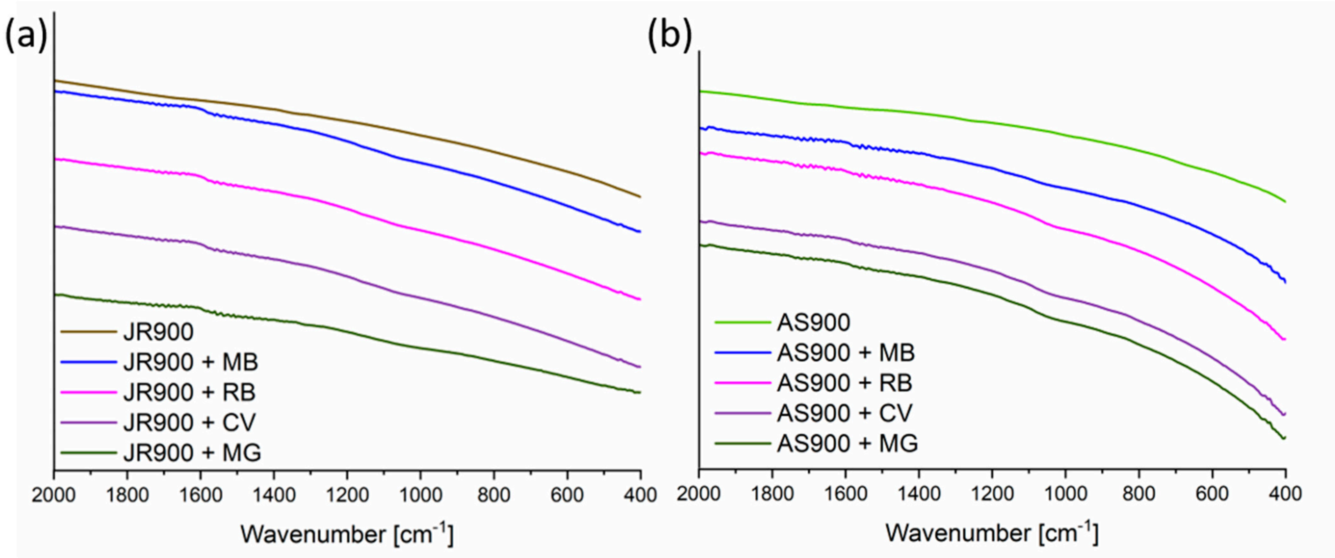

Figure S1. FTIR spectra of the adsorbents prior and after 5th cycle of adsorption and desorption

References

1. Krstić, V., *Chapter 14 - Role of zeolite adsorbent in water treatment*, in *Handbook of Nanomaterials for Wastewater Treatment*, B. Bhanvase, et al., Editors. 2021, Elsevier. p. 417-481. https://doi.org/10.1016/B978-0-12-821496-1.00024-6.
2. Saleh, T.A., *Chapter 3 - Kinetic models and thermodynamics of adsorption processes: classification*, in *Interface Science and Technology*, T.A. Saleh, Editor. 2022, Elsevier. p. 65-97. https://doi.org/10.1016/B978-0-12-849876-7.00003-8.
3. Lagergren, S., About the theory of so-called adsorption of soluble substances. **1898**.
4. Ho, Y.S.; McKay, G., Pseudo-second order model for sorption processes. *Process Biochemistry* **1999**, *34*, p. 451-465. https://doi.org/10.1016/S0032-9592(98)00112-5.
5. Roginsky, S.; Zeldovich, Y.B., The catalytic oxidation of carbon monoxide on manganese dioxide. *Acta Phys. Chem. USSR* **1934**, *1*, p. 2019.
6. Weber Jr, W.J.; Morris, J.C., Kinetics of adsorption on carbon from solution. *Journal of the sanitary engineering division* **1963**, *89*, p. 31-59.
7. Al-Ghouti, M.A.; Da'ana, D.A., Guidelines for the use and interpretation of adsorption isotherm models: A review. *Journal of Hazardous Materials* **2020**, *393*, p. 122383. https://doi.org/10.1016/j.jhazmat.2020.122383.
8. Freundlich, H.M.F., Over the adsorption in solution. *J. Phys. chem* **1906**, *57*, p. 1100-1107.
9. Langmuir, I., THE ADSORPTION OF GASES ON PLANE SURFACES OF GLASS, MICA AND PLATINUM. *Journal of the American Chemical Society* **1918**, *40*, p. 1361-1403. 10.1021/ja02242a004.
10. Temkin, M., Kinetics of ammonia synthesis on promoted iron catalysts. *Acta physiochim. URSS* **1940**, *12*, p. 327-356.
11. Wang, J.; Guo, X., Adsorption isotherm models: Classification, physical meaning, application and solving method. *Chemosphere* **2020**, *258*, p. 127279. https://doi.org/10.1016/j.chemosphere.2020.127279.
12. Dubinin, M. *The equation of the characteristic curve of activated charcoal*. in *Dokl. Akad. Nauk. SSSR.* 1947.

39. Milanković, V.; Tasić, T.; Brković, S.; Potkonjak, N.; Unterweger, C.; Pašti, I.; Lazarević-Pašti, T., Sustainable carbon materials from biowaste for the removal of organophosphorus pesticides, dyes, and antibiotics. *Journal of Environmental Management* **2025**, *376*, p. 124463. https://doi.org/10.1016/j.jenvman.2025.124463.
40. Sangsuk, S.; Napanya, P.; Tasen, S.; Baiya, P.; Buathong, C.; Keeratisoontornwat, K.; Suebsiri, S., Production of non-activated biochar based on Biden pilosa and its application in removing methylene blue from aqueous solutions. *Heliyon* **2023**, *9*. 10.1016/j.heliyon.2023.e15766.
41. Jiang, D.; Li, H.; Cheng, X.; Ling, Q.; Chen, H.; Barati, B.; Yao, Q.; Abomohra, A.; Hu, X.; Bartocci, P.; Wang, S., A mechanism study of methylene blue adsorption on seaweed biomass derived carbon: From macroscopic to microscopic scale. *Process Safety and Environmental Protection* **2023**, *172*, p. 1132-1143. https://doi.org/10.1016/j.psep.2023.02.044.
42. Li, P.; Zhao, T.; Zhao, Z.; Tang, H.; Feng, W.; Zhang, Z., Biochar Derived from Chinese Herb Medicine Residues for Rhodamine B Dye Adsorption. *ACS Omega* **2023**, *8*, p. 4813-4825. 10.1021/acsomega.2c06968.
43. Kumbhar, P.; Patil, S.; Narale, D.; Sartape, A.; Jambhale, C.; Kim, J.-H.; Kolekar, S., Biobased carbon for effective removal of rhodamine B and Cr(VI) from aqueous solution: kinetic, isotherm and thermodynamic study. *Biomass Conversion and Biorefinery* **2024**, *14*, p. 3535-3550. 10.1007/s13399-022-02625-8.
44. Raji, Y.; Nadi, A.; Mechnou, I.; Saadouni, M.; Cherkaoui, O.; Zyade, S., High adsorption capacities of crystal violet dye by low-cost activated carbon prepared from Moroccan Moringa oleifera wastes: Characterization, adsorption and mechanism study. *Diamond and Related Materials* **2023**, *135*, p. 109834. https://doi.org/10.1016/j.diamond.2023.109834.
45. Jabar, J.M.; Adebayo, M.A.; Owokotomo, I.A.; Odusote, Y.A.; Yılmaz, M., Synthesis of high surface area mesoporous ZnCl₂–activated cocoa (Theobroma cacao L) leaves biochar derived via pyrolysis for crystal violet dye removal. *Heliyon* **2022**, *8*. 10.1016/j.heliyon.2022.e10873.
46. Mosoarca, G.; Vancea, C.; Popa, S.; Dan, M.; Boran, S., Crystal Violet Adsorption on Eco-Friendly Lignocellulosic Material Obtained from Motherwort (Leonurus cardiaca L.) Biomass. *Polymers* **2022**, *14*, p. 3825.
47. Giri, B.S.; Sonwani, R.K.; Varjani, S.; Chaurasia, D.; Varadavenkatesan, T.; Chaturvedi, P.; Yadav, S.; Katiyar, V.; Singh, R.S.; Pandey, A., Highly efficient bio-adsorption of Malachite green using Chinese Fan-Palm Biochar (Livistona chinensis). *Chemosphere* **2022**, *287*, p. 132282. https://doi.org/10.1016/j.chemosphere.2021.132282.
48. Abewaa, M.; Mengistu, A.; Takele, T.; Fito, J.; Nkambule, T., Adsorptive removal of malachite green dye from aqueous solution using Rumex abyssinicus derived activated carbon. *Scientific Reports* **2023**, *13*, p. 14701. 10.1038/s41598-023-41957-x.
49. Ahmad, T.; Manzar, M.S.; Firmansyah, M.L.; Khan, S.; Ahmed, I.U.; Younas, M.; Ullah, N., Biomass-derived activated carbons from Ziziphus jujube and Dodonaea viscosa for the efficient removal of malachite green: a comparative adsorption performance and mechanisms. *International Journal of Environmental Analytical Chemistry* **2026**, *106*, p. 648-666. 10.1080/03067319.2025.2522255.
